# Transcriptional dynamics of the murine heart during perinatal development at single-cell resolution

**DOI:** 10.1242/bio.062493

**Published:** 2026-09-11

**Authors:** Lara Feulner, Florian Wünnemann, Naimeh Rafatian, Jenna Liang, Anaïs Medouni, Philipp Hofmann, Marc-Phillip Hitz, Denis Schapiro, Severine Leclerc, Patrick Piet van Vliet, Gregor Andelfinger

**Affiliations:** ^1^ Cardiovascular Genetics, Department of Paediatrics, Centre Hospitalier Universitaire Sainte-Justine Research Centre, Montreal, QC H3T 1C5, Canada; ^2^ University of Montreal, Department of Molecular Biology, Montreal, QC H3T 1J4, Canada; ^3^ Institute for Computational Biomedicine, Faculty of Medicine, University Hospital Heidelberg and Heidelberg University, 69120 Heidelberg, Germany; ^4^ McGill University, Department of Anatomy and Cell Biology, Montreal, QC H3A 0G4, Canada; ^5^ German Centre for Cardiovascular Research, Institute of Medical Genetics, Carl von Ossietzky University, 26129 Oldenburg, Germany; ^6^ Centre Hospitalier de I'Université de Montreal (CHUM) Research Centre, OPTILAB Diagnostic Laboratory, Montreal, QC H2X 0C1, Canada

**Keywords:** Heart maturation, Perinatal, Single-cell RNA-seq, H19, Endocardial development, Valve development

## Abstract

Heart maturation and remodelling during the foetal and early postnatal period are critical for the proper survival and growth of the foetus, yet our knowledge of the molecular processes involved is lacking for many cardiac cell types. To gain a deeper understanding of the transcriptional dynamics of the heart during the perinatal period, we performed single-cell RNA sequencing on foetal and early postnatal mouse hearts to establish a catalogue of 49,769 single-cell transcriptomes and used this for bioinformatics analyses. Pseudotime analyses and RNAscope fluorescence *in situ* hybridisation showed that while lncRNA H19 expression decreased over time in multiple cardiac cell types, it remained stably expressed in endocardial and valve endothelial cells. To further investigate this in a human setting, we generated human induced pluripotent stem cell (hiPSC)-derived endothelial cells and used single-cell data to develop a sorting strategy to separate endocardial from vascular endothelial cells, ensuring endothelial subtype purity. Knockdown of *H19* in hiPSC-derived endocardial cells resulted in morphological changes, decreased expression of endothelial markers, and increased expression of mesenchymal markers, consistent with the onset of endothelial-to-mesenchymal transition. Together, our data suggest a role for H19 in maintaining endocardial and valve endothelial cell states during perinatal heart maturation.

## Teaser

**Summary:** We generated a single-cell RNA-seq dataset of mouse heart development covering the perinatal period. Subsequent analysis indicates that the long non-coding RNA H19 plays a role in endocardial cell state.

## INTRODUCTION

The mammalian heart is a complex, multicellular organ responsible for blood circulation of deoxygenated blood to the lungs and oxygenated blood to the rest of the body. During early embryonic development, the heart forms from the first and second heart fields to give rise to the four-chambered heart (Anderson et al., 2014; Bruneau, 2013; Moore-Morris et al., 2018). Major structures are established around 7 weeks of gestational life in humans and about 14 days post-gestation in the mouse. Although the heart is nearly fully formed and functional at this point, it still undergoes extensive growth and cellular changes (Buckingham et al., 2005; Pervolaraki et al., 2018). The transition from foetal to neonatal life imposes several major challenges on the young heart, as the postnatal heart has to adapt to an increased workload due to the cessation of foetal blood circulation and the start of oxygenation via the lungs (Morton and Brodsky, 2016; Tan and Lewandowski, 2020). The large physiological changes imposed on the newborn heart are accompanied by molecular adaptations of the different cell types (Lai et al., 2008; Piquereau and Ventura-Clapier, 2018; Sim et al., 2015). Cardiac muscle cells transition from glycolysis to fatty acid energy metabolism to meet energy demands (Murphy et al., 2021a,b; Padula et al., 2021; Piquereau and Ventura-Clapier, 2018; Uosaki et al., 2015), and cardiac fibroblasts (CFIB) provide structural support via extracellular matrix (ECM) synthesis (Hortells et al., 2020; Wang et al., 2020). Previous studies investigated heart maturation using bulk technologies such as RNA sequencing (RNA-seq) and assay for transposase-accessible chromatin using sequencing (ATAC-seq), often with a focus on cardiomyocytes (CMs) (Quaife-Ryan et al., 2017; Sakamoto et al., 2020; Talman et al., 2018). Although these efforts uncovered important maturation signals for CMs, the non-myocyte cell populations were often neglected.

Single-cell RNA-seq (scRNA-seq) has become a powerful tool for interrogating the transcriptomes of different heart cell types (Paik et al., 2020; Yamada and Nomura, 2020). Early studies investigating heart development in the mouse discovered important transcriptomic changes across major cardiac cell types but were limited in throughput and the number of different cell types identified (DeLaughter et al., 2016; Li et al., 2016). Subsequent technological advances have enabled much higher throughput for mouse and human heart single-cell transcriptomes to study cardiac cell diversity and disease models (Asp et al., 2019; Cui et al., 2019; Litviňuková et al., 2020; Skelly et al., 2018; Tucker et al., 2020). Despite these improvements, most single-cell studies focused on models of early embryonic developmental processes during heart formation (de Soysa et al., 2019), postnatal CM maturation and regeneration (Hu et al., 2018; Kannan et al., 2021; Murphy et al., 2021b; Wang et al., 2020), or acute disease models like myocardial infarction and cardiomyopathy (Forte et al., 2020; Koenig et al., 2022; Li et al., 2019; Rizzo et al., 2023; Ruiz-Villalba et al., 2020; Tombor et al., 2021; Vafadarnejad et al., 2020; Wünnemann et al., 2025). This has left a gap in our understanding of the transcriptomic changes in the heart at the single-cell level during the perinatal window, the time just before and right after birth, which is a critical period for heart growth. Few studies have covered this period, with Feng et al. (2022) being a recent exception.

Here, we generated single-cell transcriptomes from whole mouse hearts across six time points from embryonic day (E)14.5 to postnatal day (P)7, covering the foetal-to-neonatal transition (further referred to as the perinatal period), using the droplet-based technology Drop-seq (Macosko et al., 2015). We identified distinct cardiac cell types and investigated the cell-type-specific and common temporal gene expression dynamics. We examined gene expression changes over the perinatal period and identified the long non-coding RNA (lncRNA) H19 as decreasing over time in multiple cell types, except in endocardial endothelial cells and valve endothelial cells (VECs). *H19* knockdown in human induced pluripotent stem cell (hiPSC)-derived endocardial cells resulted in reduced endothelial marker expression and increased mesenchymal gene expression, pointing towards H19 playing a role in preventing endothelial-to-mesenchymal transition (EndoMT) during perinatal heart maturation.

## RESULTS

### Single-cell diversity in the mouse heart during perinatal maturation

**Fig. 1.**
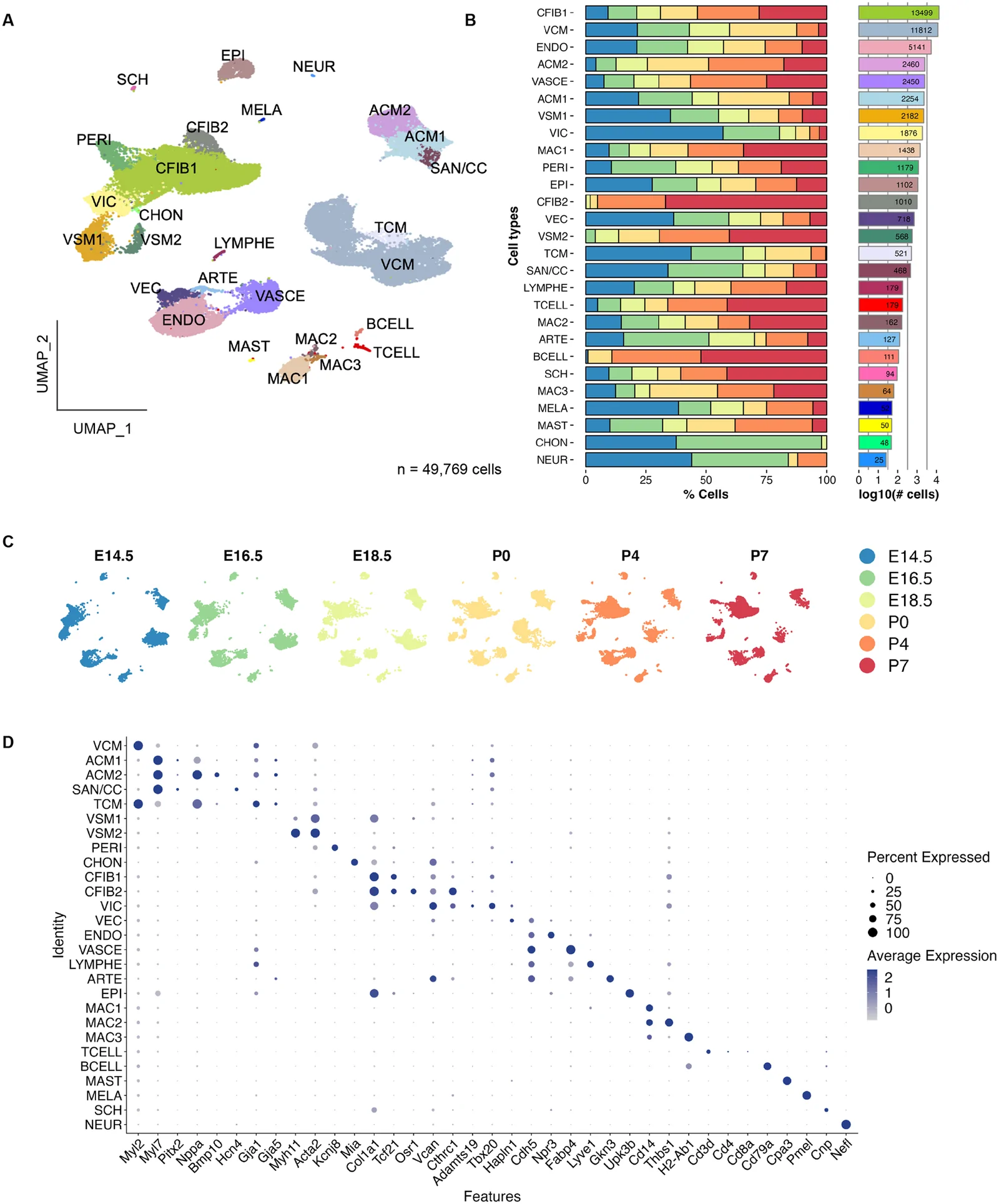
**Single-cell RNA-seq analysis of perinatal mouse hearts.** (A) Two-dimensional UMAP embedding of 49,769 cardiac cells from E14.5, E16.5, E18.5, P0, P4, and P7 mice. Six replicates were used per time point for a total of 36 different samples. Abbreviations are as follows. ACM: atrial cardiomyocyte, ARTE: arterial endothelial cell, BCELL: B cell, CFIB: cardiac fibroblast, CHON: chondrocyte (non-cardiac), ENDO: endocardial cell, EPI: epicardial cell, LYMPHE: lymphatic endothelial cell, MAC: macrophage, MAST: mast cell, MELA: melanocyte, NEUR: neuron, PERI: pericyte, SAN/CC: sinoatrial node/conduction system, SCH: Schwann cell, TCELL: T cell, TCM: trabecular cardiomyocyte, VASCE: vascular endothelial cell, VCM: ventricular cardiomyocyte, VEC: valve endothelial cell, VIC: valve interstitial cell, VSM: vascular smooth muscle cell. (B) The fraction of cells per time point across all cell types. Cell types are ordered by the total number of cells from top to bottom. Numbers within the right-side bars represent total cell count, while the *x*-axis is on a log10 scale. (C) UMAP embedding plots for individual time points. Known biological progression can be observed, for example, the lack of fibroblasts at E14.5 with extensive proliferation thereafter. (D) Dot plot of marker genes for cardiac cell types/subtypes. These were used to guide our manual cluster annotation. Abbreviations for cell types are listed above and in the main text.

We performed scRNA-seq using Drop-seq on dissociated whole mouse hearts at three foetal (E14.5, E16.5, E18.5) and three neonatal (P0, P4, P7) time points. After quality control, we obtained a final merged dataset of 49,769 single-cell transcriptomes (E14.5: 8889 cells, E16.5: 8396 cells, E18.5: 6058 cells, P0: 9213 cells, P4: 8922 cells, P7: 8291 cells) across perinatal heart remodelling and maturation stages (Fig. 1A-C; Table S1). Seurat clustering and manual annotation using known markers revealed common cardiac cell types across the six sampled time points (Fig. 1D; Fig. S1, Table S2).

CMs were an abundant cell type in our dataset (35% of all cells), consistent with previously reported relative cell quantities in the heart, including Pinto et al.’s finding that CMs comprise 32% of all cardiac cells (Banerjee et al., 2007; Pinto et al., 2016; Zhou and Pu, 2016). Three major CM subtypes, atrial (ACM; *Myl7*), ventricular (VCM; *Myl2, Myl3, Myh7*), and trabecular (TCM; *Nppa*, *Gtsf1*), were identified. Trabecular cardiomyocytes mostly resembled ventricular cardiomyocytes in their transcriptional signature but also shared several known markers with atrial cardiomyocytes such as atrial natriuretic peptide A (*Nppa*) (Houweling et al., 2005; Seidman et al., 1991). We detected two ACM subtypes: ACM1 and ACM2. Differential gene expression analysis revealed that *Pitx2*, known to regulate left atrial identity and to be postnatally restricted to the left atrium (Kirchhof et al., 2011; Tessari et al., 2008), was enriched in ACM1 (Table S3). We further noted that *Bmp10*, known to be postnatally restricted to the right atrium (Chen et al., 2004), was the second highest differentially expressed gene after *Nppa* when sorted per adjusted *P*-value. Both *Bmp10* and *Nppa* were highly enriched in ACM2. Interestingly, Pitx2 has itself been implicated in selective left atrial Bmp10 mRNA downregulation during foetal development (Tessari et al., 2008). Overall, it appears that the ACM1/2 clustering is reflective of left-right identity, with ACM1 enriched for LA and ACM2 for RA (Fig. S1, top row). High-resolution clustering additionally revealed an ACM1-like, *Nppa*-negative cluster of CMs expressing genes associated with the cardiac conduction system, such as *Hcn4*, *Vsnl1*, *Shox2*, and *Slc22a1* (Goodyer et al., 2019), likely containing cells of the sinoatrial node and the rest of the cardiac conduction system (SAN/CC) (Fig. 1D; Fig. S1).

Mural and interstitial cells represented the largest group of cells that were readily sampled at all time points (41% of cells). These cells were composed of CFIB (*Dpt*, *Tcf21*, *Lsp1, Spon2*), vascular smooth muscle cells (VSM; *Tagln*, *Acta2*, *Rgs5*), pericytes (PERI; *Kcnj8*, *Abcc9*, *Higd1b*) and valve interstitial cells (VICs; *Vcan*, *Col9a1, Prss35, Adamts19, Cthrc1*) (Litviňuková et al., 2020; Miao et al., 2024; Muhl et al., 2022; Skelly et al., 2018; Wünnemann et al., 2020). Both CFIB and VSM were subdivided into two clusters, with CFIB2 being notably enriched for *Osr1* and VSM2 being enriched for the mature smooth muscle cell marker *Myh11*. In contrast to the interstitial and mural cell populations that shared some similarity in their transcriptional profiles, epicardial cells (EPI) expressed highly specific genes (*Upk3b*, *Upk1b*, *Wt1*, *Msln*, *Krt19*) (Du et al., 2023; Knight-Schrijver et al., 2022).

Several different subtypes of endothelial cells (ECs; 17% of cells) were present in our dataset. ECs were identifiable via the canonical markers VE-cadherin (*Cdh5*) and CD31 (*Pecam1*) and were further subdivided into vascular (VASCE; *Fabp4*, *Cd36*, *Cav1*), endocardial (ENDO; *Npr3*, *Mmrn2, Emcn*), lymphatic (LYMPHE; *Ccl21a*, *Lyve1*, *Mmrn1*) and VECs (*Hapln1*, *Fxyd5*, *Lsamp, Enpp2, Cldn11*) per prior literature (Feng et al., 2019; Hulin et al., 2019; Lu et al., 2023). An additional endothelial cluster was classified as arterial (ARTE) based on strong expression of *Gkn3* (Vanlandewijck et al., 2018) in addition to *Gja4*, *Fbln5*, *Bmx*, and *Eln*.

Macrophages were the predominant immune cell subtype found among cardiac cells (Litviňuková et al., 2020; Skelly et al., 2018; Zaman and Epelman, 2022). We identified three CD14^+^ macrophage subsets: MAC1 (*C1qa*, *Mrc1*, *Pf4*, *Ccl2*), MAC2 (*Plac8*, *S100a8*, *Thbs1*) and MAC3 (*H2-Eb1*, *H2-Aa*, *H2-Ab1*, *Cd74*). Other myeloid and lymphoid cell types were seen at later developmental stages, including B cells (BCELL; *Cd79a*, *Ighm*, *Ly6d*), T cells (TCELL; *Trbc2*, *Ms4a4b*, *Cd3d*) and mast cells (MAST; *Cpa3*, *Hdc*, *Il1rl1*).

Finally, we identified small but clearly distinct clusters of melanocytes (MELA; *Pmel*, *Mlana*, *Dct*, *Plp1*), Schwann cells (SCH; *Erbb3*, *Mal*, *S100b*, *Plp1*), and neuronal cells (NEURO; *Nefl*, *Nefm*, *Elavl2*) (Litviňuková et al., 2020; Kaucka et al., 2021; Nasim et al., 2024; Skelly et al., 2018). A small additional cluster of E14.5-E16.5 cells enriched for *Col2a1*, *Mia*, and *Wif1* was identified as probable chondrocytes (CHON) and thus likely of tracheal rather than cardiac origin. These cells were therefore excluded from subsequent analysis. While most cell types were found in all biological replicates at all time points, rare cell types such as neuronal cells, melanocytes, B and T cells were sampled only in some of the biological replicates we profiled, owing to their low relative abundance in each isolated heart.

**Fig. 2.**
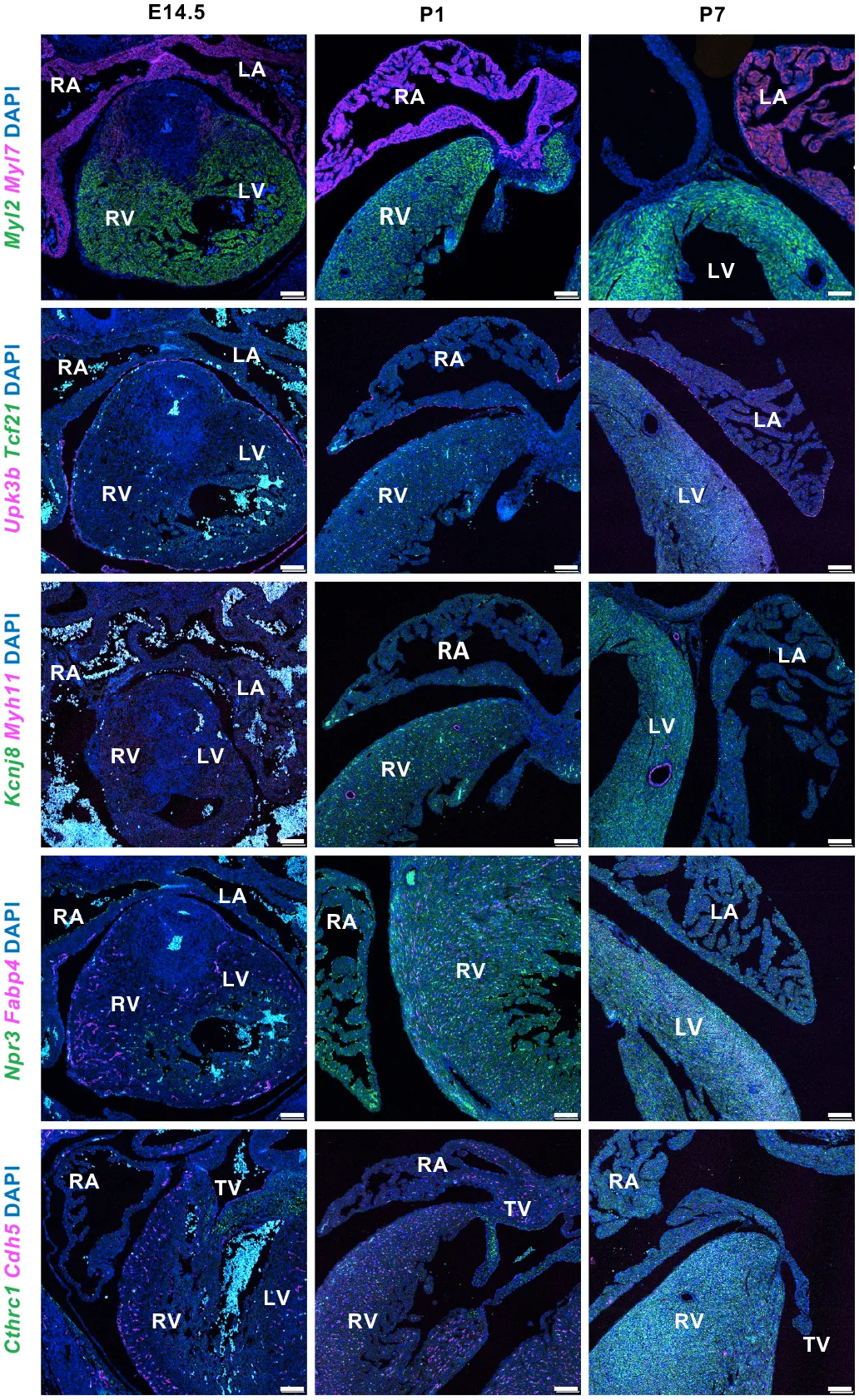
**RNAscope staining for selected marker genes across E14.5, P1, and P7 time points**. *Myl2* and *Myl7* stained for ventricular cardiomyocytes and atrial cardiomyocytes, respectively. *Tcf21* and *Upk3b* stained for cardiac fibroblasts and epicardial cells. *Kcnj8* and *Myh11* stained for pericytes and vascular smooth muscle cells. *Npr3* and *Fabp4* stained for endocardial cells and vascular endothelial cells. *Cthrc1* and *Cdh5* stained for valve interstitial cells and pan-endothelial cells. DAPI stained for cell nuclei. RA: right atrium, RV: right ventricle, LA: left atrium, LV: left ventricle, TV: tricuspid valve. Scale bars: 50 μm. Please note that images from the top row (P1/P7), second row (E14.5/P1) and fourth row (E14.5) are replicated in Fig. S2 (10×), while images from the fifth row (E14.5/P1) are replicated in Fig. S7 (10×).

To experimentally validate the cell specificity of our markers, we performed RNAscope fluorescent *in situ* hybridisation using probes for the canonical markers *Myl2*, *Myl7*, *Upk3b*, *Tcf21*, *Myh11*, *Kcnj8*, *Fabp4*, *Npr3*, *Cdh5*, and *Cthrc1* (Fig. 2; Fig. S2). RNAscope has been routinely used to validate cardiac single-cell analysis (Arduini et al., 2025; Demenego et al., 2025; Goodyer et al., 2019; Litviňuková et al., 2020; Liu et al., 2019; Lupu et al., 2020; Nomura et al., 2018), and our results were consistent with those studies. VCM, ACM, and EPI cells were especially well stained by *Myl2*, *Myl7*, and *Upk3b*, respectively. While no truly unique markers (i.e. not shared by other mesenchymal cell types) covering all time points could be found for VICs, *Cthrc1* was relatively specific to valves up to P0, after which significant *Cthrc1* expression was observed in non-VIC fibroblasts as well. We were particularly interested in *Npr3*, which prior studies had found to be an endocardial marker (Feng et al., 2019; Zhang et al., 2016). Our RNAscope showed *Npr3* to specifically outline atrial pectinate and ventricular trabecular endocardial cells without staining VECs (Fig. 2; Fig. S2). Additional bioinformatics and RNAscope analyses, along with prior literature (Zhang et al., 2016), conclusively validate *Npr3* being a marker for non-valve chamber endocardium (Fig. S3). Overall, the RNAscope staining validated the canonical markers used for annotating our scRNA-seq clusters.

### Cell-type-specific gene expression dynamics during cardiac foetal-to-neonatal transition

We next aimed to determine the genes involved in driving heart maturation during perinatal development. We utilised a semi-supervised approach termed psupertime (Macnair et al., 2022), which used known time-series labels (in our case, E14.5, E16.5, E18.5, P0, P4, P7) as input to assign cells a quantitative pseudotime score. The use of pseudotime was important, as not all cells progress through development at the same rate. An ordinal logistic regression model identified genes for each cell type showing differential expression across the pseudotime trajectories during foetal-to-neonatal transition. Genes with a non-zero beta coefficient were considered by psupertime to be relevant to the pseudotime ordering of the cells and thus to significantly vary in expression over time (also referred to as ‘dynamic’ expression).

Our analysis identified known isoform switches from beta (*Myh7*)- to alpha (*Myh6*)-myosin heavy chains, as well as the switch from foetal to adult Troponin I genes (*Tnni1* to *Tnni3*), in murine ventricular cardiomyocytes, consistent with previous observations (Bedada et al., 2014; DeLaughter et al., 2016; Siedner et al., 2003) (Fig. S4A). These positive control genes indicate that our approach confidently identified known, biologically relevant signals with temporal dynamic expression. We applied the same strategy to all major cell types, excluding those with inconsistent sampling across time or very low cell counts (Table S4).

The number of dynamically expressed genes per cell type varied significantly, ranging from 27 (LYMPHE) to 1556 (CFIB1) (Table S4, abs_beta>0). Fibroblasts and atrial/ventricular cardiomyocytes had by far the most genes with dynamic expression over pseudotime, potentially reflecting a greater degree of perinatal changes compared to other cell types. Fibroblasts are known to extensively proliferate postnatally and are involved in ECM remodelling, while CMs undergo significant changes, including hypertrophic cell growth with concomitant cell cycle arrest, sarcomeric protein isoform switching, and a transition to aerobic respiration (Padula et al., 2021; Uscategui Calderon et al., 2023).

**Fig. 3.**
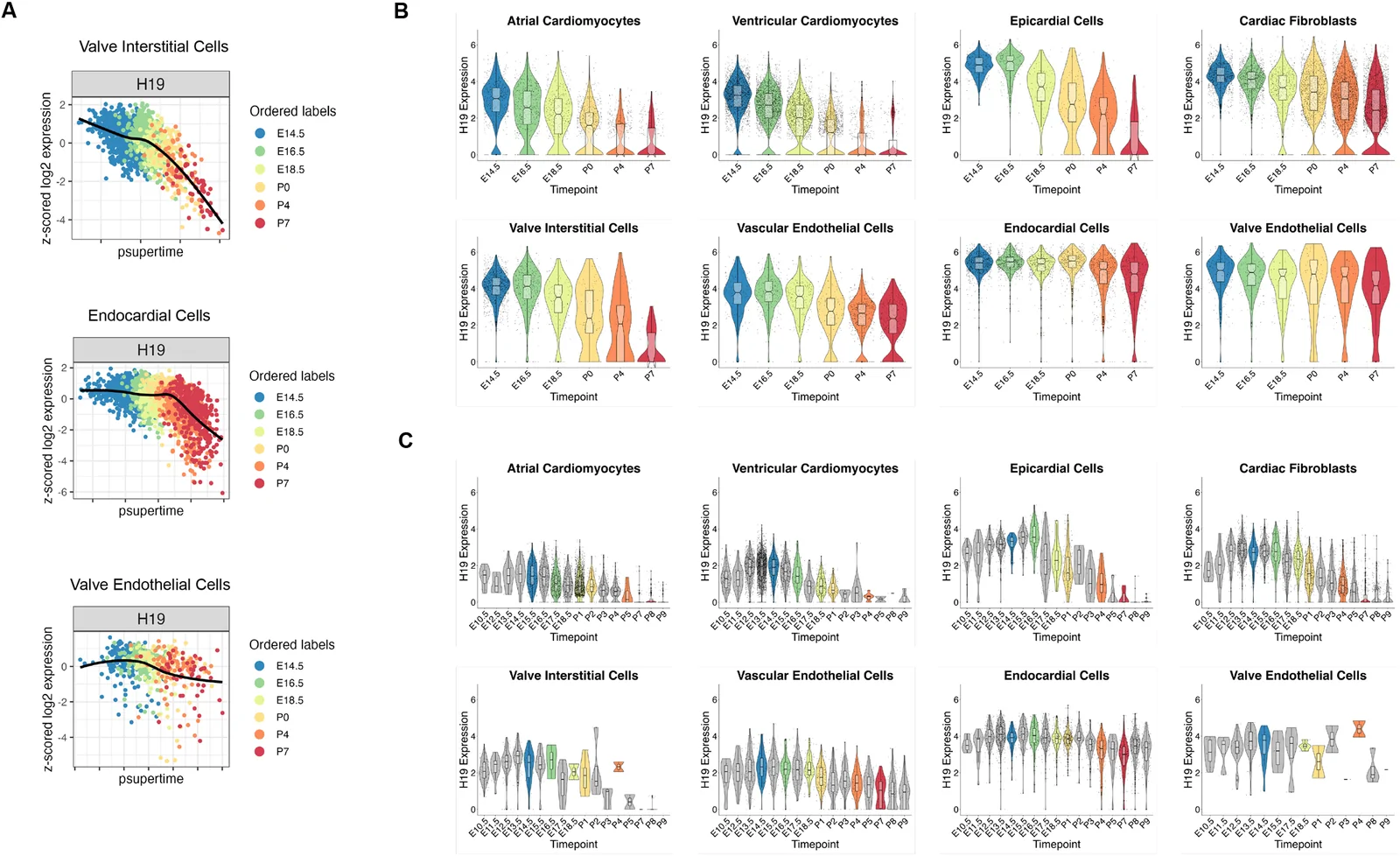
**H19 expression patterns during the perinatal period in cardiac cell types.** (A) Plots of gene expression for *H19* against pseudotime scores learned by psupertime in valve interstitial, endocardial, and valve endothelial cells. *x*-axis: pseudotime score for each cell, *y*-axis: *z*-scored log2 gene expression values. All cells are ordered by pseudotime score and coloured by their known time point so as to easily visualise changes in gene expression across time. H19 expression decreases in valve interstitial cells but is relatively maintained in endocardial cells, particularly valve endothelial cells. (B) Seurat violin plots of *H19* gene expression per time point in multiple cardiac cell types. *H19* decreases in most cardiac cells during perinatal maturation but maintains expression specifically in endocardial and valve endothelial cells. (C) Seurat violin plots as above from the public dataset by Feng et al. (2022; GSE193346). This dataset spanned a wider developmental time period. *H19* initially increases early in development, peaks around E12.5-E14.5, and decreases thereafter in most cell types but again is maintained in endocardial and valve endothelial cells.

On average, we detected 404 genes with dynamic expression over pseudotime per cell type. Of the 4886 genes identified in total, 3737 (76%) were unique to one cell type, and 234 (4.8%) were present in three or more cell types. The latter included mitochondrial genes, ribosomal genes, solute carrier (Slc) proteins, genes associated with metabolic pathways such as cyclooxygenase (Cox) and fatty acid binding protein (Fabp) family members, and some sarcomere genes. Low-level expression of *Mlc2a*, *Nkx2-5*, and *cTnt* was previously observed in the pro-epicardial organ during development (Filipczyk et al., 2007; Mommersteeg et al., 2007) and could help explain the sarcomere expression in epicardial cells. Genes with high variance in expression over time across diverse cell types included *H19* (nine cell types), *mt.Nd1* (nine cell types), *Calm1* (eight cell types), and *Cd9* (seven cell types) (Table S4). Of those, the lncRNA H19 was the top psupertime hit (based on absolute beta coefficient scores) for EPI, VASCE, VSM, and MAC and in the top five for ACM, CFIB, and VIC (Fig. 3; Fig. S4B).

### Decrease in lncRNA H19 expression is a common feature of cardiac cell maturation, with the exception of endocardial cells

Given the psupertime results, we further examined H19 expression patterns during cardiac cell maturation. H19 expression decreased strongly in most cell types, of distinct origins, from E14.5 to P7. Interestingly, endocardial cells (including VECs) were the notable exception to this pattern, showing relative maintenance of H19 expression (Fig. 3A,B). To validate this finding, we additionally analysed a recently published, independent dataset (Feng et al., 2022), which was generated using a different single-cell technology called MULTI-seq and included an expanded series of time points from E10.5 to P9. We found that the H19 dynamics in this dataset were consistent with ours. In most cases, an initial increase in H19 expression peaking around E14.5 and decreasing thereafter could be seen. Consistent with our data, H19 expression was substantially maintained in endocardial cells and VECs (Fig. 3C). We additionally analysed male and female samples separately to verify that this pattern was not sex dependent (Fig. S5).

We furthermore used a more recent independent computational tool, Sceptic (Li et al., 2025), to generate pseudotime scores in a supervised manner. Unlike psupertime, Sceptic uses a nonlinear support vector machine rather than an ordinal logistic regression model to generate pseudotime scores. When *H19* expression was plotted against Sceptic pseudotime scores, the resulting plots were notably comparable to those from our psupertime analyses (Fig. S6). We additionally used Shapley additive explanations (SHAP), which is a method commonly used to explain predictions made from machine learning models, to identify which genes most influenced the model calculating the assigned pseudotime scores. When sorted by absolute mean SHAP scores, *H19* was the top hit for EPI, VIC and VSM and top three for ACM, CFIB and VASCE. It was not, however, within the top ten for ENDO, consistent with the relatively stable H19 expression in these cells over time (Fig. S6). We note that *Tnni1* and *Tnni3* were also identified as highly influential genes in ACM and VCM, confirming that our analyses are consistent with known biological processes as well as our original psupertime analysis.

**Fig. 4.**
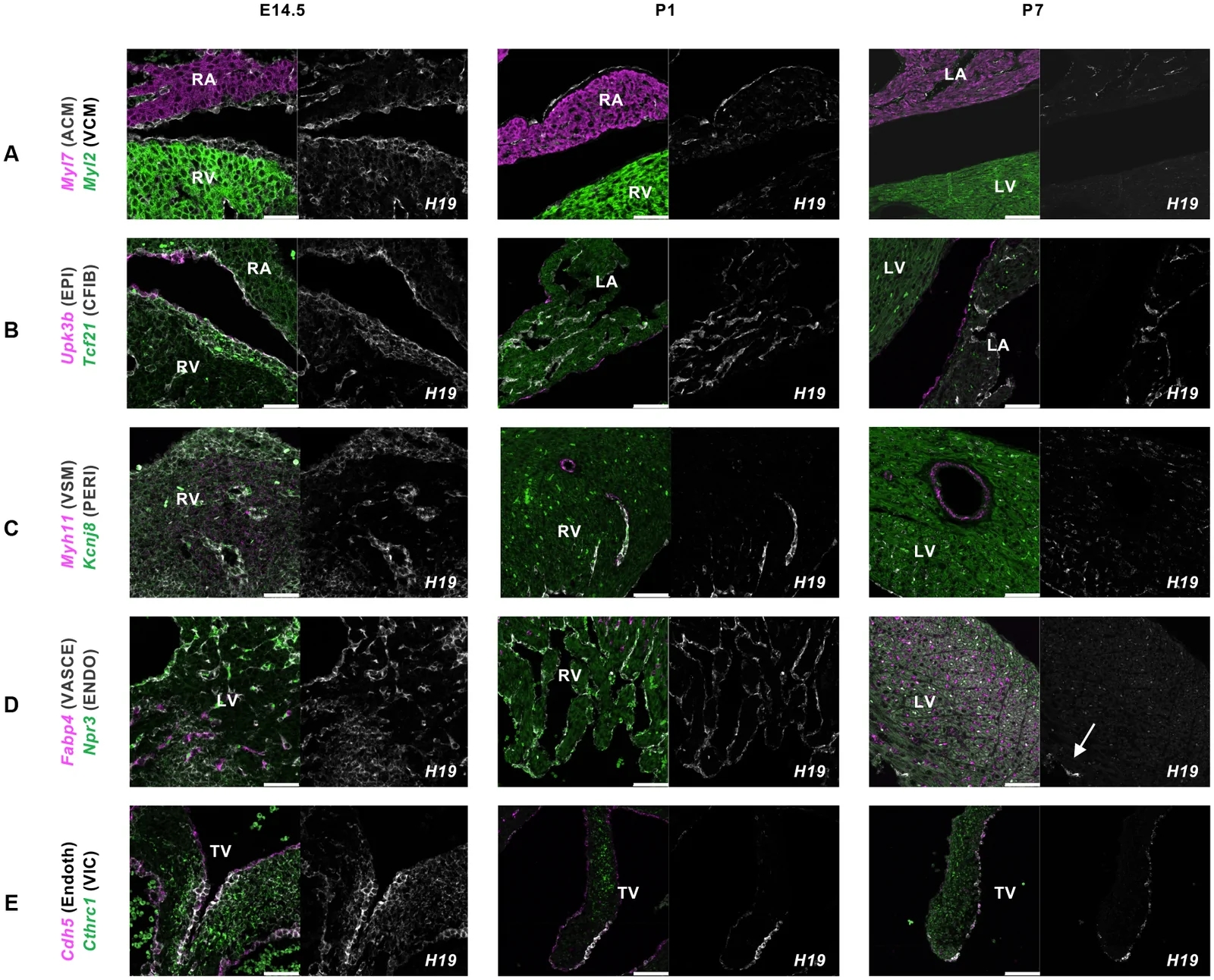
**RNAscope stains across E14.5, P1, and P7 time points for *H19* and the same cell type marker genes as in Fig. 2.** (A-E) At E14.5, H19 expression (white) is relatively high and is co-expressed with all eight cell type markers. A decrease in H19 expression is consistently seen thereafter, with the largest drop occurring from P1 to P7. Note that the remaining expression of *H19* at P7 corresponds to endocardial and valve endothelial cells, consistent with apparent maintenance seen in our scRNA-seq data. In particular, the *H19* signal in the endocardium of the left atrium at P7 can be seen in the images co-stained for *Tcf21* and *Upk3b* (B) in addition to the images from *Npr3* and *Fabp4* co-staining (D, with arrows indicating the highest intensity signal). RA: right atrium, RV: right ventricle, LA: left atrium, LV: left ventricle, TV: tricuspid valve. Scale bars: 50 μm. Please note that images from E (E14.5 and P1) are replicated in Fig. S7 (40×).

To experimentally validate these *in silico* results, we performed RNAscope staining for *H19* alongside our validated cell-type-specific markers (Fig. 4). At E14.5, *H19* expression was generally diffuse but most highly colocalised in endocardial cells and VECs, as expected from our scRNA-seq analysis. In each case, a decrease in *H19* was seen over time in non-endocardial cells, with the greatest drop occurring from P1 to P7. Notably, at P7, *H19* no longer colocalised with *Myl2* (Fig. 4A), *Upk3b* (Fig. 4B), *Myh11* (Fig. 4C), or *Cthrc1* (Fig. 4E). *H19* appeared to remain only in endocardial cells (Fig. 4B,D) and VECs (Fig. 4E), confirming maintenance of expression.

Interestingly, the RNAscope analyses showed spatially distinct valve *H19* expression over time. At E14.5, *H19* was diffusively expressed in VICs and present in VECs on both sides of the valve leaflets (Fig. 4E; Fig. S7). However, at P1 and P7, *H19* appeared to be more highly expressed in VECs at the coaptation interface.

### Shared expression dynamics highlight imprinting as an important regulator of perinatal growth

Having identified H19 as a potential regulator during heart maturation, we investigated potential downstream effects of H19 and other genes showing dynamic expression during the perinatal period. We performed enrichment analysis of 1149 genes identified by psupertime as showing significant dynamic expression in at least two different cell types, using the Mouse Genome Informatics (MGI) Mammalian Phenotype 2021 library. This analysis revealed significant (Benjamini-Hochberg adjusted *P*-value<0.05) enrichment for 37 terms (Table S5). The top three terms in order of descending *P*-value were cardiac hypertrophy (MP:0001625), increased heart weight (MP:0002833), and dilated heart left ventricle (MP:0002753), with many of the remaining significant terms referencing dilation or hypertrophy. While these terms reference pathological processes in adult hearts, the genes driving these results can also be necessary for normal perinatal heart development. A number of cardiac developmental genes are, in fact, known to correlate with hypertrophic pathology when improperly reactivated, a process referred to as the ‘foetal gene program’ (Kuwahara et al., 2012; Olson and Schneider, 2003).

A search for H19 within the Enrichr results found it to be associated with decreased body weight (MP:0001262) (*P*=0.013). It was also associated with increased placenta weight (MP:0004920), paternal imprinting (MP:0003123) and genetic imprinting (MP:0003121), all of which had high-ranking enrichment scores (combined *P*-value/odds ratio). The latter terms reflect that H19 is known for being part of the major imprinted H19/IGF2 locus, with *H19* being expressed exclusively from the maternal allele and *IGF2* from the paternal allele. Maternal imprinting (MP:0003122) was itself significant (*P*=0.03) in the enrichment analysis. We therefore reviewed the psupertime expression patterns of imprinted genes from both sets and found that VECs showed remarkably less change in expression in many of these genes compared to other cardiac cell types, such as epicardial cells, fibroblasts, and VCMs (Fig. S8). Together, this suggests that maintenance of imprinted genes may be of particular importance during perinatal endocardial and VEC maturation.

### Investigating the role of H19 in hiPSC-derived endocardial cells

The maintenance of *H19* expression in endocardial cells and VECs, but not other cardiac cell types, pointed towards a cell-specific role for H19 in the endocardium. We initially noted that there appeared to be a relatively decreasing number of endocardial cells in G2/M and S versus an increasing number of endocardial cells in G1 between E14.5 and P7 (Fig. S9). In contrast, in vascular endothelial cells, the number of cells in G2/M and S remained relatively high, and cells in G1 remained comparatively low. Based on these observations and previously reported links between H19 and cell proliferation (Guo et al., 2022; Han et al., 2016; Rigaud et al., 2023; Wang et al., 2016; Yu et al., 2021), we hypothesised that the endocardial-specific *H19* expression maintenance could play a role in regulating endocardial cell proliferation during perinatal heart maturation. However, examination of known proliferation markers *Mki67* and *Top2a* in our scRNA-seq data or immunostaining for Ki67 at E14.5, P1, and P7 was inconclusive (Fig. S10). We therefore investigated other potential cell-specific roles for H19. Notably, a prior study found increased EndoMT-transitioning cells in postnatal H19-deficient mice (Park et al., 2021). Another study found that overexpression of *H19* in human retinal ECs prevented glucose-induced EndoMT in a mechanism implicating TGFβ (Thomas et al., 2019). We therefore speculated that H19 could be involved in the maintenance of the endocardial cell state, potentially by inhibiting EndoMT.

**Fig. 5.**
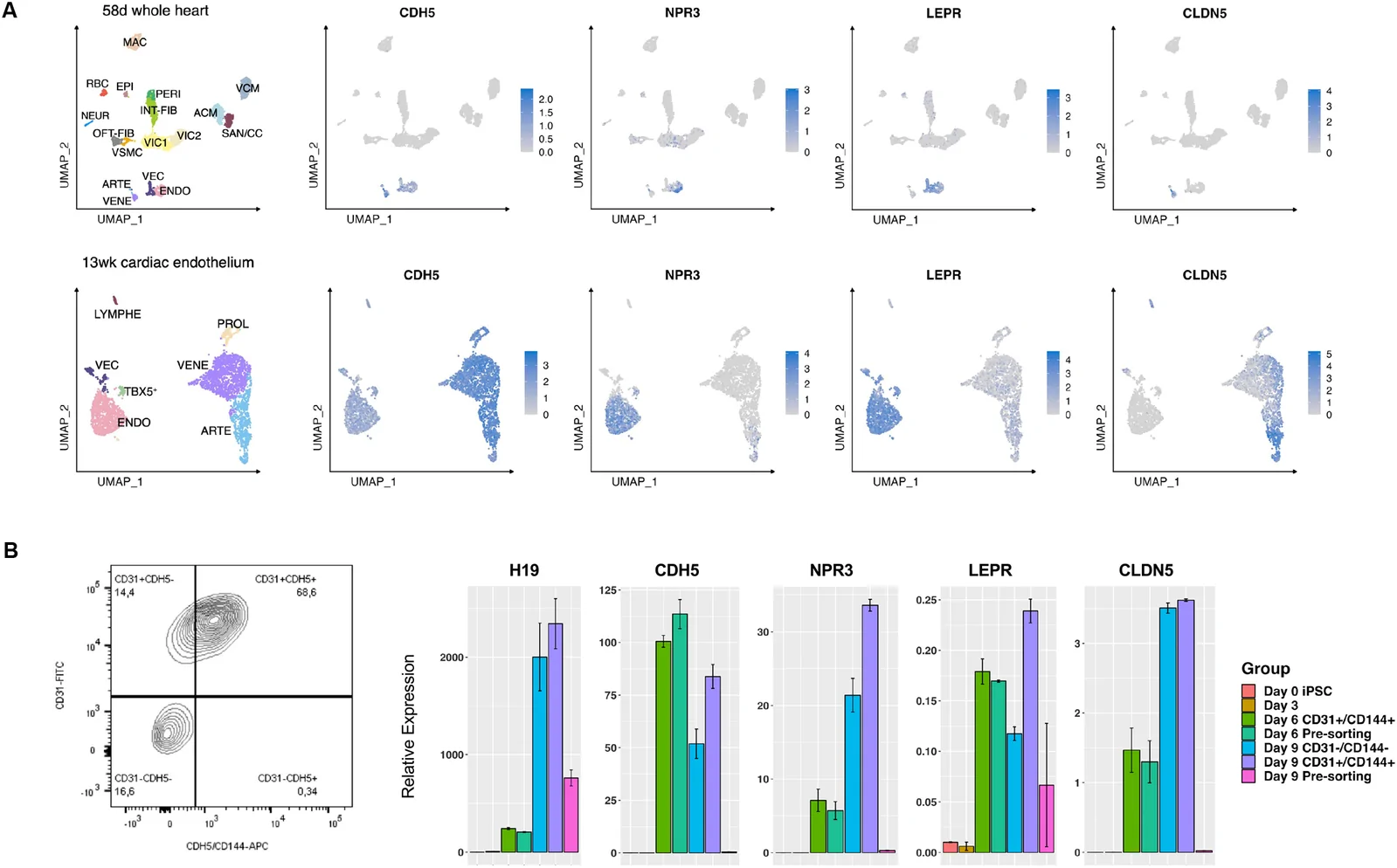
**Characterization and sorting of cardiac endothelial cell subtypes.** (A) LEPR and CLDN5 can be used as cell-surface markers to sort endocardial and vascular cells. Top row: UMAP from a reanalysed single-cell dataset from Knight-Schrijver et al. (2022) (58-day human foetal whole heart) followed by respective feature plots for CDH5, NPR3, LEPR, and CLDN5. Bottom row: same as above, using a reanalysed dataset from McCracken et al. (2022) (13-week human foetal endothelial cells pre-sorted for CDH5). LEPR is highly expressed in endocardial cells, while CLDN5 is expressed in vascular endothelial cells but absent in endocardial cells. (B) hiPSC-derived valve endothelial cell protocols generate mixed endothelial-subtype populations. Left: flow cytometry results on day 6 of differentiation following the endocardial cell protocol from Cai et al. (2024); 68.6% of cells were double positive for CD31 and CD144 and could be confidently considered endothelial. Note that all CD144^+^ cells were also CD31^+^. Right: RT-qPCR analysis for hiPSC-generated pan-endothelial cells. Bars indicate expression of CDH5, NPR3, LEPR, and CLDN5 relative to reference gene HPRT1. H19 was highly expressed following endothelial induction and reached very high levels by day 9. CDH5 expression was consistently high. Importantly, expression of both LEPR and CLDN5 was noted, indicating a heterogeneous endothelial cell population.

To investigate an endocardium-specific role for H19, we aimed to perform siRNA inhibition of *H19* in endocardial cells derived from hiPSCs. We tested various protocols to generate hiPSC-derived endocardial cells (Cai et al., 2024; Neri et al., 2019; Palpant et al., 2017) and found that the protocol from Cai et al. was the most efficient in generating ECs, with up to 60-70% double-positive for CD31^+^/CD144^+^ (PECAM1^+^/CDH5^+^) (Fig. 5B; Fig. S11). However, similar protocols were used by others for the generation of pan-ECs (Olmer et al., 2018; Patsch et al., 2015; Rosowski et al., 2023; Wimmer et al., 2019), raising the possibility that protocols intended for endocardial cell generation could generate a mix of endothelial subtypes. Since vascular endothelial cells did not appear to show the same maintenance of H19 expression as endocardial cells in our single-cell analysis of perinatal mouse heart generation, this could skew an endothelial subtype-specific interpretation of H19 function *in vitro*. We therefore set out to identify cell-surface markers that could be used to sort the two endothelial subpopulations via fluorescence-activated cell sorting (FACS).

Given that our differentiation used hiPSCs, we began examining human foetal datasets. We reanalysed datasets of 58-day foetal cardiac cells (Knight-Schrijver et al., 2022) and 13-week CDH5^+^-sorted cardiac ECs (McCracken et al., 2022). Our human public dataset reanalysis identified subtype-specific expression for *PDGFB*, *A2M*, *FGD4* and *PREX2* in human vascular endothelial cells, and *ART4*, *IL18R1* and *TMEM108* in human endocardial cells, in addition to previously known markers *NPR3* and *ADGRG6* (Table S6). For subtype-specific FACS, we focused on the cell-surface leptin receptor (LEPR), which was strongly and differentially expressed in endocardial cells (including VECs) compared to vascular endothelial cells, and the tight junction protein Claudin-5 (CLDN5), which was strongly expressed in vascular endothelial cells but essentially absent in endocardial cells (Fig. 5A). This signature was also present in prior sequencing of hiPSC-derived cells from a separate endocardial protocol (Fig. S12) (Mikryukov et al., 2021), where the authors notably identified LEPR as a putative endocardial-specific marker. We further confirmed these distinct patterns in data from human cardiac spatial transcriptomics studies (Fig. S13) (file S14 in Farah et al., 2024; file S5 in Lázár et al., 2025), which were consistent with studies in mice, where *Npr3* and *Cldn5* were shown to mark endocardial and vascular endothelium specifically (D'Amato et al., 2022). A reverse-transcription quantitative PCR (RT-qPCR) analysis of our hiPSC-derived CD31^+^/CD144^+^ pan-ECs confirmed the expression of *CDH5*, *NPR3*, *LEPR*, and *CLDN5*, consistent with endothelial subtype heterogeneity (Fig. 5B). We therefore concluded that we could separate hiPSC-derived pan-ECs into endocardial versus vascular endothelial cells based on LEPR and/or CLDN5 expression for subsequent H19 studies.

### H19 knockdown results in decreased endothelial gene state and initiation of EndoMT

**Fig. 6.**
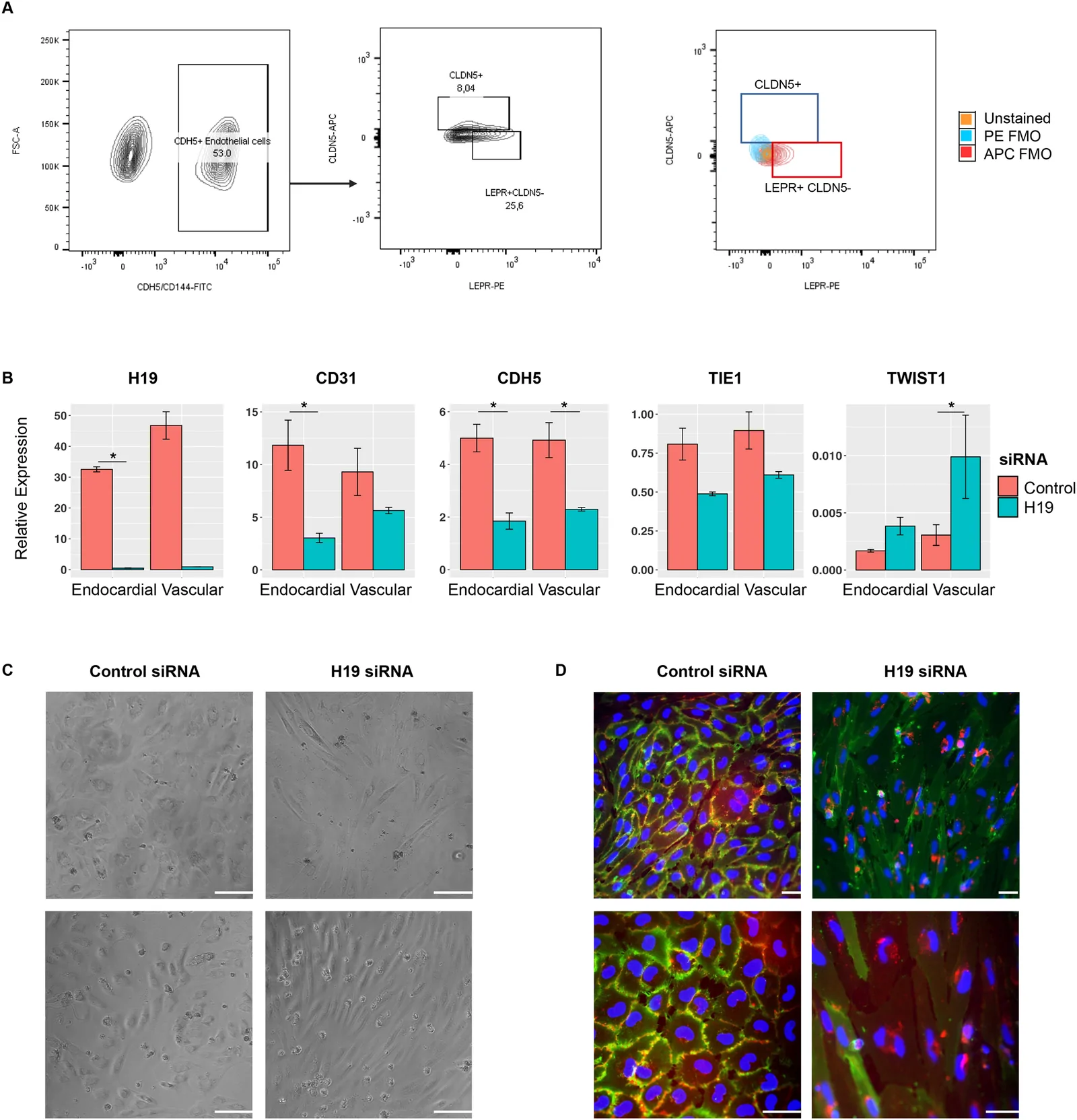
**H19 inhibits EndoMT in hiPSC-derived endothelial cells.** (A) Flow cytometry sorting for endocardial and vascular endothelial cells. Pan-endothelial cells were initially sorted by gating for CD144 (CDH5). The CDH5^+^ cells were then sub-selected for LEPR^+^/CLDN5^−^ endocardial cells and CLDN5^+^ vascular endothelial cells. The rightmost panel shows the fluorescence-minus-one (FMO) control gating settings. (B) RT-qPCR results for hiPSC-derived cells sorted for CD144^+^/CLDN5^−^ (endocardial) and CD144^+^/CLDN5^+^ (vascular endothelial). For each bar plot, the *y*-axis represents the relative expression of the respective gene of interest vs the geometric mean of reference genes HPRT1 and RPL13A. Salmon-coloured bars represent cells transfected with control siRNA, and aqua/teal-coloured bars represent cells transfected with H19 siRNA. CD31 RNA expression decreased notably in H19 siRNA-transfected CLDN5^−^ endocardial cells, but a smaller decrease was observed in CLDN5^+^ vascular endothelial cells (*n*=2; mean±s.d.; *P<0.05, unpaired two-tailed *t*-test). (C) Brightfield imaging of hiPSC-derived endothelial cells showing morphological changes in H19 siRNA-transfected cells. The more oblong spindle-shaped appearance is associated with a mesenchymal phenotype. Top row: CLDN5^−^ cells (endocardial), bottom row: CLDN5^+^ cells (vascular endothelial). 10× magnification. Scale bars: 200 μm. (D) Immunofluorescence of hiPSC-derived CLDN5^−^ endocardial cells showing decreased CD31 protein expression (green) in H19 siRNA-transfected cells. CDH5 (red) is no longer expressed at the cell membrane. Top row: 20× magnification (scale bars: 100 μm), bottom row: 40× magnification (scale bars: 50 μm).

hiPSC-derived CD31^+^/CD144^+^ cells were sorted into CLDN5^−^ endocardial and CLDN5^+^ vascular populations (Fig. 6A), replated, and transfected with control or H19 siRNA. RT-qPCR showed that cells transfected with control siRNA have high *H19* expression in both populations, whereas transfection with H19 siRNA showed near-complete knockdown of *H19* (Fig. 6B). H19 siRNA-transfected cells also showed decreased expression of EC markers *CD31*, *CDH5* and *TIE1* as compared to controls. Conversely, while expression was low, an increase could be seen in *TWIST1*, a transcription factor highly implicated in EndoMT (Fig. 6B). Cells from both endocardial and vascular populations transfected with H19 siRNA underwent morphological changes and took on a spindle-shaped appearance more associated with mesenchymal cells (Fig. 6C). Immunofluorescence staining of endocardial cells confirmed decreased CD31 and perturbed CDH5 protein expression upon H19 knockdown (Fig. 6D). Altogether, this suggests that H19 knockdown leads to early EndoMT in both endothelial subtype populations.

## DISCUSSION

In this study, we performed high-throughput, droplet-based single-cell transcriptomics of mouse hearts at six different time points throughout the perinatal period (E14.5 to P7). We identified major cell types such as CFIBs and CMs but were also able to sample much less frequent cells, such as MELAs and NEUROs. This allowed us to determine the common transcriptional response of cardiac cells during the transition from foetal to neonatal life. The overall top time-varying gene identified by psupertime was the lncRNA H19, part of a major imprinting locus with IGF2. *H19* decreased during perinatal cardiac maturation across multiple cell types in our analysis as well as in an independent dataset, and this was further verified experimentally via RNAscope.

The H19/IGF2 locus has been well studied and is known to be involved in foetal programming through DNA methylation (Szabó et al., 2004). In general, IGF2 has a growth-promoting effect while H19 has a growth-restraining effect. H19 was in fact highlighted as anti-hypertrophic in a study where ectopic induction of H19 expression was sufficient to suppress CM hypertrophy *in vitro* (Viereck et al., 2020). Another study positively linked cardiovascular pathology in mouse loss-of-imprinting models to misexpression of H19 and reduction of H19 binding to Mirlet7 (Park et al., 2021). Misregulation of the H19/IGF2 locus causes growth disorders in humans, including Beckwith-Wiedemann syndrome (BWS) and Silver-Russell syndrome (SRS) (Bliek et al., 2006; Sparago et al., 2004). Congenital heart disease has been reported in conjunction with BWS/SRS (Ghanim et al., 2013; Greenwood et al., 1977). This is consistent with findings from +/H19^hIC1^ (+/hIC1) SRS mouse models, where the imprinting control region (ICR) of H19/Igf2 is mutated on the paternal allele, leading to overexpression of *H19* and depletion of *Igf2*. In addition to placental defects, +/hIC1 mice showed atrioventricular (AV) cushion defects by E12.5, followed by incomplete interventricular septum formation, ventricular septal defects, and perinatal lethality (Chang et al., 2022). Interestingly, these defects persisted following normalisation of *H19* expression alone (via maternal deletion of *H19*) and were rescued only by normalisation of both *H19* and *Igf2* expression (via maternal deletion of the H19/Igf2 ICR), demonstrating that proper dosage of both genes is required for normal cardiac and placental development. Given the importance of endocardial cells in AV cushion formation and cardiac septation, RNA-seq of CD31+ cardiac ECs was performed, indicating dysregulation of pathways related to proliferation and ECM production in +/hIC1 versus wild-type mice (Chang et al., 2022).

In our study, endocardial cells appeared to be an exception to the observed overall downregulation of H19, instead showing relative maintenance of *H19* expression. The majority of heart valve development takes place between E9.5 and E14.5 (Feulner et al., 2022). Previous reports suggested that subsequent endocardial contributions are restricted by a VEC-specific Nfatc1 enhancer (Zhou et al., 2005). Nfatc1 modulates EndoMT in a cell-autonomous manner by suppressing transcription of *Snail1/2* and promoting transcription of *Cdh5* (Wu et al., 2011). This is relevant as the interaction of H19 with the polycomb repressive complex 2 suppresses H3K27 tri-methylation of the Tescalcin locus, leading to reduced NFAT expression and activity (Viereck et al., 2020). H19 may thus play a role in limiting Nfatc1 signalling via the autoregulated enhancer in VECs, ensuring normal balance between EndoMT and valve elongation. This would be consistent with further evidence suggesting that H19 plays a role in EndoMT. Park et al. found increased EndoMT-transitioning cells (co-expressing endothelial CD31 and mesenchymal αSMA/SM22α markers) in postnatal H19-deficient mice as compared to wild type. Furthermore, H19 rescue transfection of H19-deficient P2 ECs reduced expression of SM22α as well as transcription factors (Snail and Slug) critical for EndoMT (Park et al., 2021). H19 was also found to prevent EndoMT in retinal endothelium via TGFβ (Thomas et al., 2019) and to specifically inhibit TGFβ2-induced EMT of the eye lens by suppressing Smad-dependent signalling (Xiong et al., 2022). By contrast, H19 was found to induce EndoMT via let-7 inhibition and TET1 activation in human umbilical vein ECs (Cao et al., 2020). H19 regulation of EndoMT is thus likely context dependent (Busscher et al., 2022). Our findings that endocardial H19 expression is maintained during perinatal heart maturation beyond E14.5 suggest that maintenance of the endocardial cell state may be an ongoing process.

To study the effect of H19 perturbation and potential impact on EndoMT in specific hiPSC-derived endothelial subtypes (hiPSC-ECs), we used single-cell data to develop a strategy to sort endocardial endothelial cells from vascular endothelial cells using LEPR and CLDN5 as markers. LEPR was chosen over the previously described NPR3 strategy (Miao et al., 2020) since our analyses showed that NPR3 is not expressed in mouse perinatal (Fig. S3) or human foetal VECs (Fig. 5A). While CLDN5 was previously identified as a vascular rather than endocardial marker (D'Amato et al., 2022), our study validates its vascular specificity in hiPSC-ECs. This new strategy will be of benefit to future studies in hiPSC-derived endothelial subtypes since our tests of various directed endothelial differentiation protocols revealed the generation of a heterogeneous pan-endothelial mix. Upon sorting and H19 transfection in hiPSC-derived endothelial subtypes, RT-qPCR showed highly efficient *H19* knockdown. Subsequently, there was a decrease in RNA expression of endothelial markers *CD31*, *CDH5* and *TIE1*, with a corresponding increase in the EndoMT marker *TWIST1*. Decreased CD31 and perturbation of CDH5 protein expression were further confirmed by immunofluorescence. *H19*-knockdown cells, furthermore, took on a more mesenchymal, spindle-shaped appearance. Altogether, this pointed towards H19 siRNA-transfected cells undergoing early EndoMT, prior to the appearance of mature mesenchymal markers.

While we found minor differences in H19 siRNA-induced changes in endothelial and mesenchymal marker expression in endocardial versus vascular endothelial cells, we did not observe substantial differences at the morphological level between these subtypes in their response to *H19* knockdown. This is likely due to the hiPSC-EC immaturity, with cells being more equivalent to mouse foetal than postnatal ECs. The results do, however, demonstrate that H19 prevents EndoMT onset, consistent with prior studies (Park et al., 2021; Thomas et al., 2019; Xiong et al., 2022). We therefore propose that stable *H19* expression in mouse endocardial cells (which remained high even at P7) contributes to maintenance of the endocardial state and that misregulation of H19 might lead to aberrant EndoMT. Further experimental validation is required to determine whether H19 and Nfatc1-mediated regulation of VEC phenotypes and perinatal valve maturation are related.

In contrast to the endocardial cells, downregulation of *H19* in other cardiac cell types is likely required for the continuous contributions of these cell types to early postnatal cardiac growth. H19 overexpression in VICs has been found to inhibit the NOTCH1 pathway, leading to increased expression of RUNX2 and the development of an osteoblastic VIC phenotype resulting in calcific aortic valve disease (CAVD) (Hadji et al., 2016). Conversely, knockdown of H19 increased NOTCH1 and decreased RUNX2. Since Notch gain-of-function mouse mutants develop VIC-like cells in the non-valve endocardium (MacGrogan et al., 2018), it would be interesting to determine if H19 acts via Notch signalling to prevent aberrant EndoMT in the chamber endocardium.

Overall, our study suggests that modulation of imprinted genes, in particular the lncRNA H19, is important for cardiac cell maturation. The downregulation of *H19* observed in many cell types, including VICs, may play a hitherto-unappreciated role in maintaining normal proliferative levels and cell states, with disease implications in cases of dysregulation. Simultaneously, our results suggest that stable H19 expression contributes to phenotypic maintenance of the mouse endocardial cell state.

### Limitations

Our study validated known cardiac cell types and markers, and we additionally identified novel candidate genes and pathways involved in the transition from foetal to neonatal life. However, our analyses (and those in other scRNA-seq studies requiring tissue dissociation) may be affected by capturing CMs at a lower rate with increasing age, while less fragile cells, such as stromal and interstitial cells, are relatively enriched. Consequently, maturing binucleated or multinucleated CMs may be underrepresented. Another factor hindering capture of maturing CMs is their increasing size, which can make them too big to be captured in microfluidics-based single-cell applications. However, these limitations were likely minor, given that our cell ratios were consistent with those reported in previous non-scRNA-seq studies (Banerjee et al., 2007; Pinto et al., 2016; Zhou and Pu, 2016).

We noted stronger expression of *H19* in human foetal heart vascular endothelial cells compared to endocardial and valve ECs (Fig. S14). This is in contrast to our mouse scRNA-seq data, where *H19* was most highly expressed in endocardial cells. Similarly, we noted slightly higher *H19* expression in control hiPSC-derived vascular endothelial cells versus endocardial cells (Fig. 6B). However, these species-specific differences did not necessarily prevent our human-mouse comparisons. Similar examples can be found with respect to X-chromosome inactivation and reactivation (XCI/XCR, controlled by the lncRNA Xist), for which there are notable differences in the mechanisms between mice and humans to achieve X-chromosome dosage compensation (Petropoulos et al., 2016). Past attempts to replicate XCI/XCR *in vitro* found that human embryonic stem cells (hESCs) derived from pre-implantation blastocysts did not display the X-chromosome state of human pre-implantation epiblast cells *in vivo* and instead shared molecular and functional properties with mouse post-implantation epiblasts (Khan et al., 2017). Furthermore, we examined supplemental output from bulk RNA-seq of a separate protocol deriving endocardial cells from hiPSCs (file S1 in Liu et al., 2023). Their BMP10-derived endocardial cells had a 4.3 log2FC higher expression of *H19* compared to VEGF-derived vascular endothelial cells. By comparison, *NKX2-5*, the primary marker cited in the study as distinguishing the two populations, had a log2FC of 3.7. This suggests that *H19* was indeed enriched in these hiPSC-derived endocardial cells, consistent with our *in vivo* mouse scRNA-seq data. The high *H19* expression in both of our hiPSC-derived endothelial subtypes, which were derived with the Cai et al. protocol (Cai et al., 2024), is consistent with the mouse foetal *H19* expression, where the differences between endocardial endothelial cells and vascular endothelial cells are less pronounced. Together, this suggests that our hiPSC-derived ECs are more similar to foetal than postnatal mouse ECs. Further optimisation and comparisons of directed differentiation protocols will be needed in the future to resolve these species-specific and/or protocol-dependent differences.

## MATERIALS AND METHODS

### Animal ethics and collection of heart tissue

All experiments involving mice were performed according to the Canadian Council on Animal Care (CCAC) guidelines and were approved by the ethical committee of the CHU Sainte-Justine Research Centre. Mice acquired from The Jackson Laboratory (C57Bl/6NJ, stock nr.: 005304) were maintained on a C57Bl/6NJ background at the CHU Sainte-Justine animal facility. Mice were kept on a 12-h light/dark cycle (06:00 to 18:00) under specific-pathogen-free (SPF) conditions, with *ad libitum* access to water and food on a standard chow diet (2918 Teklad Global 18% protein rodent diets, Envigo). Room temperature was maintained between 20°C and 24°C with 40-70% humidity, according to CCAC guidelines. Mouse crosses were set up between wild-type C57Bl/6NJ mice. Matings were checked in the morning, and if a copulation plug was present, 12:00 of that day was counted as embryonic day (E)0.5 of the pregnancy.

Methods for extracting foetal hearts were the same as in Wünnemann et al. (2020). Briefly, pregnant females were sacrificed by injection of pentobarbital followed by cervical dislocation. The uterus containing foetuses was extracted and kept on ice in Dulbecco's PBS (DPBS) during dissection. Fine dissection of individual foetuses was performed in cold DPBS under a stereoscopic microscope. For postnatal time points, the morning of birth was considered as postnatal day (P)0, and pups were processed the same day, while P4 and P7 were processed 4 and 7 days following the morning of birth, respectively. For all time points, the heart was extracted by cutting the descending aorta using scissors and carefully pulling out the lungs together with the connected heart. Any remaining lung tissue, trachea, fat or non-cardiac tissue was removed. The pulmonary veins and the inferior vena cava were cut as close to the base of the heart as possible, and the pulmonary artery and aorta were cut close to the pulmonary valve and aortic valve, respectively, to exclude the majority of these two large vessels from the tissue dissociation.

### Single-cell dissociation of heart tissue

Dissected hearts were cut into small pieces using fine dissecting scissors and transferred to gentleMACS C Tubes (Miltenyi Biotec, Cat-Nr.: 130-093-237) containing enzymatic digestion solution according to the manufacturer's protocol for the Neonatal Heart Dissociation Kit (Miltenyi Biotec, Cat-Nr.: 130-098-373). Two mouse hearts were processed in separate C tubes at the same time for all experiments presented in this manuscript. The C tubes were subsequently fitted onto a gentleMACS Octo Dissociator (Miltenyi Biotec, Cat-Nr.: 130-095-937) using the program *37C_mr_NHDK_1*, which performs enzymatic and gentle mechanical tissue dissociation at 37°C according to the manufacturer's protocol. Following completion of the dissociation program, C tubes were briefly centrifuged at 400 ***g*** to collect all liquid and cells from the lid and walls of the tube. Cells were then treated according to the manufacturer's protocol. Briefly, 7.5 ml of DMEM high-glucose medium without phenol red (Thermo Fisher, Cat-Nr.: 21063029) containing 10% FBS preheated to 37°C was added to the cells, followed by resuspension by mild pipetting (five to ten times using a 10 ml pipette). Cells in DMEM+FBS were subsequently filtered through a 70 μm filter (Miltenyi Biotec, Cat-Nr.: 130-098-458) and transferred to 15 ml conical Falcon tubes. Filters were washed with 3 ml of warm DMEM+FBS.

Following filtration, cells were pelleted for 5 min at 600 ***g*** at room temperature. The supernatant was removed, and cell pellets were resuspended in 1 ml of PEB buffer (DPBS pH 7.4, 0.5% BSA, 2 mM EDTA) followed by the addition of 10 ml of 1× red blood cell (RBC) lysis buffer (Miltenyi Biotec, Cat-Nr.: 130-094-183). Cells were incubated in RBC lysis buffer for 2 min at room temperature and then centrifuged at 600 ***g*** for 5 min at room temperature. The supernatant was removed, and cell pellets were resuspended in 10 ml DPBS+15 μl of Enzyme A of the dissociation kit. Cells were then centrifuged for a final time at 600 ***g*,** and the resulting pellets were resuspended in 1 ml of DPBS+0.01% BSA for counting. Cells were counted using a disposable Fuchs-Rosenthal haemocytometer (INCYTO C-Chip disposable haemocytometer) with Trypan Blue to assess viability. Following counting, cells were diluted to a final concentration of 140 to 160 cells/μl and processed using Drop-seq.

### Single-cell RNA-seq of mouse hearts using Drop-seq

Dissociated cells were captured in nanolitre droplets using a custom Drop-seq set-up at the CHU Sainte-Justine Research Center in the Andelfinger laboratory. The custom Drop-seq set-up and protocol closely followed the originally published protocol at https://mccarrolllab.org/dropseq/ (Macosko et al., 2015) with a few minor differences. We utilised Smith's Medical Medfusion 2500 pumps in place of KD Scientific Legato 100 pumps as described in the original article. Cell dissociations were run through polydimethylsiloxane (PDMS) chips ordered with Aquapel treatment from FlowJEM. To capture single-cell transcriptomes, cells were run alongside droplet generation oil (Bio-Rad, Cat-Nr.: 186-4006) and barcoded beads (ChemGenes, CSO-2011), which were suspended in cell lysis buffer (see the original Drop-seq protocol) supplemented with 50 μl of RNase inhibitor (SUPERase·In, Cat-Nr.: AM2694) per millilitre of lysis buffer. The barcoded beads contain oligos with a bead-specific cell barcode, an oligo-specific unique molecular identifier (UMI) and a polyT sequence to capture polyadenylated RNA. Cells and beads were run at speeds of 4 ml h^−1^, while oil was run at 15 ml h^−1^. During each Drop-seq run, the aqueous flows from 1 ml of beads and cells were collected. Following droplet capture, droplets were diluted in 30 ml of 6× saline-sodium citrate (SSC; Thermo Fisher, Cat-Nr.: AM9763) and broken by shaking after adding 1 ml of perfluorooctanol (PFO; Fisher Scientific, Cat-Nr.: AAB2015618). Beads were spun down and washed twice in 6× SSC and once in a home-made reverse transcriptase (RT) buffer. Beads were resuspended in the RT mix (the same reagents as the original Drop-seq protocol) and subjected to reverse transcription for 30 min at room temperature followed by 90 min at 42°C in a rotating hybridisation oven. After reverse transcription, beads were washed and treated with Exonuclease I for 45 min at 37°C with rotation. Following exonuclease treatment, beads were washed and counted using a Fuchs-Rosenthal haemocytometer in a 1:1 ratio with bead counting solution (10% PEG, 2.5 M NaCl, as described by Gierahn et al. 2017).

To amplify single-cell transcriptomes, 4000 beads [∼200 single-cell transcriptomes attached to microparticles (STAMPS)] were used as input for individual PCRs, as described in the original Drop-seq protocol. Ten PCRs were performed in parallel, for a total of 2000 STAMPs per biological sample. PCRs were pooled post-PCR for the desired final number of STAMPS in the library and cleaned up twice using AMPure XP beads at 0.6× concentration. The quantity and size distribution of cleaned cDNA were checked using the Bioanalyzer High Sensitivity Assay (Agilent Genomics, Cat-Nr.: 5067-4626). Libraries for sequencing were prepared from 600 pg cDNA input using a Nextera XT library preparation kit (Illumina, Cat-Nr.: FC-131-1096) with i7 primers from Illumina (N70X) together with a custom P5 primer (see the Drop-seq protocol). Quality control of libraries was performed using the Bioanalyzer High Sensitivity Assay. Libraries were quantified and quality controlled using the Universal Library Quantification Kit (KAPA) and were sequenced on an Illumina NextSeq 500 at the Institute for Research in Immunology and Cancer (IRIC) in Montreal. Read settings for sequencing were R1: 20 bp, R2: 63 bp, index: 8 bp.

### Processing and annotation of single-cell RNA-seq data

Raw Illumina sequencing data was demultiplexed using bcl2fastq (v2.17). FASTQ files for E14.5 are publicly available (GSE150817; Wünnemann et al., 2020) and can be downloaded from the sequence read archive (SRA) using the SRA explorer (https://sra-explorer.info/). Raw FASTQ files for each sample were processed using kallisto and bustools (kb-python, v0.24.4) against the ensemble reference release 98 for *Mus musculus* (GRCm38, primary assembly; see the Colab notebook) (Melsted et al., 2021). We utilised emptyDrops from the DropletUtils package in R to call cells from the raw, unfiltered matrices from kb-python (FDR threshold≤1%) (Lun et al., 2019). Genes expressed in less than five cells per sample were subsequently filtered out. To detect potential cell doublets, UMI matrices filtered based on emptyDrops were analysed per time point, using the scrublet package (v.0.2.3) in R via reticulate with default parameters and an expected doublet rate of 5% to calculate doublet scores. We subsequently removed cells with a scrublet doublet score over 0.25 as predicted doublets.

After sample-level QC filters, sample matrices were merged into one joined Seurat object. We then applied general QC filters, removing cells with more than 10k UMIs and with less than 500 total genes detected. To exclude stressed and dying cells, we also excluded cells with more than 15% mitochondrial gene expression. After quality control steps, we ended up with a set of 68,912 cells that we used for a first round of clustering. We utilised SCTransform (Seurat 4.0.0) via the SCTransform function in Seurat to normalise UMI counts for sequencing depth (Hafemeister and Satija, 2019). We included the percentage of mitochondrial gene expression, cell cycle, and sex as variables to regress out in the SCTransform normalisation and scaling step. For calculating cell cycle scores for regression, we translated human gene symbols from Nestorowa et al. (2016) to mouse gene symbols using the Bioconductor biomaRt R package (Durinck et al., 2005) and subsequently assigned cell cycle scores and predicted cell cycle phase for all cells. We used S and G2M scores for each cell in the SCTransform step from Seurat to remove potential cell cycle effects from the dimensional reduction. After SCT normalisation, principal components (PCs) were calculated in Seurat based on the 3000 most highly variable genes. We used 50 PCs for Uniform Manifold Approximation and Projection (UMAP) embedding of all cells into two dimensions (McInnes et al., 2020 preprint). Unsupervised cell clusters were identified using a shared-nearest-neighbour (SNN) graph, followed by the original Louvain clustering algorithm as implemented in Seurat FindNeighbors() and FindClusters() functions.

Potential marker genes in clusters assigned by Seurat were calculated using the wilcoxauc() function from the presto package (Korsunsky et al., 2019 preprint). After the first round of clustering, we removed additional cell clusters from the dataset. First, we noted one cluster of cells that mostly consisted of cells from one sample and expressed high levels of genes not generally expressed in the heart but in the lungs [marked by *Wfdc2* (Bingle et al., 2020 preprint) and *Scgb3a2* (Naizhen et al., 2019)]. These cells likely present contamination from non-cardiac tissue during dissection and were therefore removed from the final dataset. We further identified a cluster that was grouped with CMs in high-dimensional space but expressed a mix of many different cell type markers and showed low UMI content and high percentage of mitochondrial gene expression. We classified these to either be damaged cells, multiplets or empty droplets with high ambient RNA content and therefore manually removed them. Finally, two clusters represented foetal and postnatal RBCs [marked by gamma-globin (*Hbb-y*) and haemoglobin subunit epsilon-Y2 (*Hbb-bs*), respectively] that remained after RBC lysis. Since RBCs were not of interest in this study, we excluded them from the final dataset. There were 67,330 cells remaining following the initial filter.

A second, more stringent round of QC was subsequently performed using parameters specific to each time point and, in some cases, replicate, with the goal of eliminating remaining outliers. In particular, it could be seen that while a mitochondrial cut-off of 15 was often appropriate for postnatal cells, a lower cut-off was more optimal for earlier cells. Overall, E14.5 cells were filtered for 500-2500 genes, 1000-5000 counts and percent mito 0.5-5, while P7 cells were filtered for 500-2000 genes, 1000-8000 counts and percent mito 0.5-8/15. This additional filtering resulted in a final dataset of 49,769 cells covering six time points (each with six replicates), from which we ran SCTransform normalisation again on raw UMI counts and re-calculated the clustering to compute final cell embeddings. Our final clustering was derived using RunUMAP with dims 1:83 and FindClusters at various resolutions ranging from 0.3 to 10. Stable marker genes for major cell types across the sampled time points were calculated using Seurat's FindMarkers() function using the RNA slot of the SCT assay with min.pct=0.1, min.diff.pct=0.1, and only.pos=T.

### Subsetting and reclustering of cell types

Each respective cell type was subsetted from the final Seurat dataset and converted to raw data using the DropletUtils package, which was then re-clustered to verify cell type purity and manually remove any outlying noncorresponding (e.g. *Myl2*-containing) clusters before continuing analysis. For each cell type, PCA DimPlots grouped by time point were examined and used to guide whether to merge subtypes for further downstream analysis. Because CFIB2 and VSM2 populations were primarily postnatal and appeared to form different trajectories on PCA plots (Fig. S15), CFIB1 and VSM1 clusters alone were used for analysis of fibroblast and smooth muscle populations. ACM1 and ACM2 populations, however, appeared to form a more continuous trajectory. This could be due to fewer transcriptional differences between the two subtypes throughout maturation, possibly indicating cell state changes, as opposed to the more substantial changes in fibroblasts or smooth muscle subtypes. This is consistent with further analysis indicating the two populations reflected left/right identity. Therefore, we merged ACM1 and ACM2 for further analysis. MAC1-3 were likewise merged. While there was some separation between the three macrophage populations, we reasoned that this cell type was sufficiently distinct from the rest and thus combining them would not skew the downstream results.

### Pseudotime analysis of single-cell data

To identify genes that are relevant for the progression of cells from E14.5 to P7, we used ordinal logistic regression as implemented in https://github.com/wmacnair/psupertime (Macnair et al., 2022) and followed a similar approach as presented by Cook and Vanderhyden (2020). We first estimated pseudotime using standard log-normalised UMI counts (RNA@data slot) from the Seurat object as input for psupertime. Pseudotime was calculated separately for each cell type that had at least ten cells per time point sampled and cells identified in at least five of six time points. This filter excluded neuronal cells, B cells and T cells from the time series analysis. psupertime was run with penalisation=‘best’, testing all genes identified by Seurat in the given cell type. The result was an R psupertime object in which the genes showing significant dynamic change in expression over time were contained (specifically, psuper_object$beta_dt$symbol). These genes could be shown using the function plot_identified_genes_over_psupertime.

For Sceptic analysis, our original E14.5-P7 time labels were assigned a real-time value in number of days post-gestation (14, 16, 18, 20, 24 and 27). Sceptic's ‘run_sceptic_and_evaluate’ command was run in Python on normalised data, using the same subsetted cell types as for psupertime with the real-time labels. Plots of *H19* expression versus pseudotime were generated with Plotnine (Python equivalent to ggplot2 in R). Note that LOWESS (locally weighted scatterplot) smoothing was performed since the GAM (generalised additive model) method was unavailable in Plotnine; otherwise, the coding replicated psupertime's own plot function. The resulting pseudotime values were then used as input for SHAP analysis. A model was first trained to predict gene expression from pseudotime using ‘train_test_split’ from scikit-learn and XGBRegressor from XGBoost. This model was then passed on to shap.explainer, and a bar plot was generated of the resulting absolute SHAP values for each cell type.

Enrichment testing for a common maturation signature was performed on genes that were significantly differentially expressed in more than two different cell types against the Enrichr (Chen et al., 2013; Kuleshov et al., 2016; Xie et al., 2021) MGI Mammalian Phenotype Level 4 2021 library at https://maayanlab.cloud/Enrichr.

### Public data analysis

#### Mouse data

A processed Seurat object provided by Feng et al. (GSE193346_C57BL6_seurat_object.rds) was downloaded from GSE193346. Cell types were isolated by subsetting for clusters enriched for cell-specific markers. In the case of VICs and VECs, the cluster was further subset to only include cells from the left ventricle corresponding to the described methods in the paper.

#### Human data

FASTQ files were downloaded from the European Nucleotide Archive (ENA; https://www.ebi.ac.uk/ena/browser/home) onto Alliance Canada servers for accession numbers SRR21953763+SRR21953764 (Knight-Schrijver et al., 2022) and SRR17848279 (McCracken et al., 2022). The FASTQ files were subsequently uploaded to the 10x Genomics Cloud server for processing with Cell Ranger v9.0.0 using GRCh38 2024-A as a reference.

The resulting count matrices were then processed using Seurat v4. For QC, SoupX (Young and Behjati, 2020) was used to remove ambient RNA, and the package scDblFinder (Germain et al., 2021) was used to remove doublets. Normalisation and scaling were performed using Seurat's SCTransform function while regressing for cell cycle in the 58-day foetal heart data. PCA, UMAP generation and clustering were subsequently performed.

For spatial data (Lázár et al., 2025), an interactive viewer was accessed at https://hdcaheart.serve.scilifelab.se. Genes of interest were viewed for ‘section_1 (8 weeks)’. In addition, R objects related to the study were downloaded from https://data.mendeley.com/datasets/w65jtfsvpr/1, and the original UMAPs were used to generate feature plots for ECs. We thank the authors for providing these files.

For hiPSC-derived endothelium (Mikryukov et al., 2021), an SRA file corresponding to accession number GSM4568142 (SRX8399633, run SRR11849413) was downloaded from Gene Expression Omnibus (GEO; https://www.ncbi.nlm.nih.gov/geo) and converted to FASTQ using fasterq_dump before processing as described above.

### RNAscope

Preparation of samples and pre-treatment were performed according to the instructions in the RNAscope Multiplex Fluorescent Reagent Kit v2 user manual. C57BL/6NJ hearts were collected and fixed overnight for paraffin embedding. Paraffin sections of 5 μm thickness were heated for 1 h at 60°C. Paraffin was removed from the slides by two Histo-Clear washes for 5 min, washed twice in 100% ethanol for 5 min, and briefly dried at 60°C. Slides were incubated with hydrogen peroxide for 10 min at room temperature, and a manual target retrieval (Appendix B of the protocol) was performed for 15 min at 95°C, and then the slides were left to dry. RNAscope Protease Plus was added for 30 min and incubated at 40°C in the HybEZ Humidifying System Oven (Advanced Cell Diagnostics). Slides were washed twice for 2 min with distilled H_2_O. The probes were heated to 37°C for 30 min and cooled down to RT. One volume of C2, C3, and/or C4 probes was added to 50 volumes of C1 probe or TSA buffer. The probe mixes were incubated for 2 h at 40°C, and the slides were washed twice for 2 min with wash buffer. All probes used were manufactured by Advanced Cell Diagnostics for the Manual Assay RNAscope and included the following: Adamts19 (500981; C4), Cdh5 (312531; C1), Cthrc1 (413341; C4), Fabp4 (884621; C2), H19 (423751; C3), Hapln1 (448201; C2), Kcnj8 (411391; C1), Myh11 (316101; C2), Myl2 (527951; C2), Myl7 (584271; C1), Npr3 (502991; C2), Tcf21 (508661; C1), Upk3b (568561; C1). All ensuing incubations were performed at 40°C in the HybEZ oven; RNAscope wash buffer was used between each step. Incubation for AMP1 and AMP2 was 30 min, followed by AMP3 for 15 min. The horseradish peroxidase (HRP) was developed by using the RNAscope Multiplex FL V2 HRP-C1, HRP-C2, HRP-C3, and/or HRP-C4 reagent for 15 min followed by incubation with an opal dye (Opal 570, FP1487001KT, Perkin Elmer; Opal 620, FP1488001KT, Perkin Elmer; or Opal 690, FP1497001KT, PerkinElmer). The HRP signal was blocked by adding the RNAscope Multiplex FL V2 blocker for 15 min. After washing, slides were counterstained with DAPI for 1 min at RT and mounted with a fluorescence mounting medium (S3023; Dako). Fluorescence was observed with a SP8-DLS confocal microscope (Leica Microsystems). Images were taken with the 10× or 20× objective without or with 2× zoom (final 40× magnification) and a total Z-size of 12 μm (number of steps: 7; Z-step size: 2 μm). Image maximum projection was done with LAS X imaging software (Leica, version 3.3.0.16799).

### Stem cell culture and VEC differentiation

The use of hiPSCs was approved by the ethics committee at the participating institution, CHU Sainte-Justine, Montreal, QC, Canada (CER# 2287, MP-21-2006-107). hiPSCs were generated at the CR-CHUSJ Stem Cell facility. The hiPSC line SJ3013C2 was derived from human fibroblasts of a healthy 40-year-old Quebecois male (Liu et al., 2021; Song et al., 2024). hiPSCs were maintained in mTeSR Plus (STEMCELL Technologies #100-0276, #100-1130) on plates coated with Corning^®^ Matrigel^®^ hESC-Qualified Matrix (Cat.-Nr.: 354277) and passaged with Gentle Cell Dissociation Reagent (STEMCELL Technologies #100-0485, 100-1077) when they reached 70% confluency.

To differentiate cells into VECs, we used the protocol published by Cai et al. (Cai et al., 2024). Briefly, hiPSCs were dissociated into single cells using Accutase (STEMCELL Technologies #07920) and then plated onto a Matrigel-coated plate at a concentration of 30,000 cells/cm^2^ in mTeSR Plus supplemented with 5 μM Y-27632 (Selleck #S1049). The next day, directed differentiation was started by replacing media with differentiation media containing a 1:1 mixture of neurobasal medium (Life Technologies, 21103049) and DMEM/F-12 (Life Technologies, 11320-033), with 1.94% B27 minus vitamin A (Life Technologies, 12587010), 0.97% N2 (Life Technologies, 17502048), 8 μmol/l CHIR-99021 (Selleck, S2924), and 25 ng/ml BMP4 (PeproTech, 120-05). Cells were maintained in this medium for 3 days, after which the medium was replaced with StemPro-34 SFM medium (Gibco, 10639011) supplemented with 200 ng/ml VEGF-165 (PeproTech, 100-20) and 1 μmol/l forskolin (Selleck, S2449). Cells received freshly supplemented media every day. On day 6, cells were dissociated using 2× TrypLE Select, prepared from 10× (Gibco, A1217701), for sorting the EC population.

For initial characterisation, cells were strained on a filter, blocked with 5% FBS in PBS, and stained with 1:25 FITC-labelled CD31 (BD Biosciences, Cat-Nr.: 555445, PRID AB_395838) and 1:25 APC-labelled CD144 (BioLegend, Cat-Nr.: 348508, PRID AB_1063994). Samples were sorted with a 100 μm nozzle in two-way purity mode with a BD FACSAria Fusion instrument (BD Biosciences, San Jose, CA, USA) for CD31/CD144-double-positive ECs. To separate endocardial and vascular endothelial cells for H19 experiments, the cells were triple-labelled for 1:25 CD144-FITC (BioLegend, 376510), 1:25 LEPR-PE (RD System, FAB867P), and 1:100 CLDN5-APC (AntibodySystem, FHA14113). First, pan-ECs were selected by gating for CD144. These pan-ECs were then sub-selected for LEPR^+^/CLDN5^−^ endocardial cells versus CLDN5^+^ vascular endothelial cells. Fluorescence minus one (FMO) was used to set the gating. Endocardial and vascular endothelial cells were replated and cultured in EGM2 medium (Lonza, CC-3202).

### Real-time PCR

RNA was extracted using ReliaPrep™ RNA Miniprep (Promega). The extracted RNA was reverse transcribed using SuperScript IV (Invitrogen, 18090050) and random hexamers (Invitrogen, N8080127). Real-time qPCR was performed on a CFX Opus 96 (Bio-Rad) PCR system to quantify the expression levels of various genes and reference genes using the primers described in Table S7 and FastStart Universal SYBR Green Master (Rox) (Roche #4913850001). The delta-Ct method was used to quantify gene expression. The results shown in Fig. 6B are representative of one hiPSC differentiation batch with two biological replicates from wells on separate plates (*n*=2). For each biological replicate, the delta-Ct of two technical replicates was averaged. An unpaired two-tailed *t*-test was used to assess significance (*P*<0.05) using the default t_test command from the rstatix package in R.

### H19-transfected cells

H19 antisense (ref. 4392420, Invitrogen) or negative control antisense (ref. 4390843, Invitrogen) at a concentration of 10 pmol in OPTI-MEM (Invitrogen, 51985034) was mixed with FuGENE^®^ SI Transfection Reagent (Promega, E9311) at a final concentration of 5 μl/ml of OPTI-MEM. OPTI-MEM solution containing antisense and transfection reagent was mixed and added to EGM2 medium, which was then added to the cells plated in a Matrigel-coated 48-well plate. Two days after transfection, the media were changed. On day 5 post-differentiation, cells were either collected for RNA extraction or PFA fixed for immunofluorescence analysis.

### Immunofluorescence

Cells were fixed with 4% PFA in PBS, washed, and blocked with 5% goat serum (EMD Millipore, 50024585) and 1% BSA in PBS. Cells were labelled with 1:100 CD31 (RD system, AF806-SP) and 1:25 Alexa Fluor^®^ 647 Mouse Anti-Human CD144 (BD Biosciences, Cat-Nr.: 561567, PRID AB_1071276) overnight at 4°C. CD31 was labelled with 1:1000 donkey anti-sheep IgG (H+L) cross-adsorbed 488 secondary antibody (Thermo Fisher Scientific, A-11015). DAPI-counterstained cells were imaged using a wide-field microscope (Leica DMi8).

### Data analysis

Data analysis and statistical tests in this work were performed using R version 4.2.3 in RStudio (≤2023.06.0+4.1) (©RStudio Inc.) and Python (version 3.8.13) in Jupyter Notebook.

### Data and code availability

Raw FASTQ files and processed, joined data matrices of all final cells used in the paper are available at NCBI GEO (GSE150817). Raw data for E14.5 hearts were published previously (GSE109247) (Wünnemann et al., 2020). We selected six wild-type replicates (GSM2935824, GSM2935825, GSM2935826, GSM2935827, GSM3677518, and GSM3677519) from the E14.5 dataset for equal number of replicates across all time points. Our data have also been uploaded to Chan Zuckerberg CELL by GENE Discover for visual exploration. Code scripts and files are available at https://github.com/larafeulner/Perinatal_Mouse_Heart_scRNAseq. Additional files are hosted on Zenodo at https://zenodo.org/records/20650169.

## Supplementary Material

10.1242/biolopen.062493_sup1 Supplementary information

Table S1. Metadata information for all cells in final single-cell dataset. Fastq files for E14.5 samples were previously published (Wünnemann et al.) and reanalysed from raw data using the same pipeline used for all other samples. Six replicates were used per time point for a total of 36 mice as indicated in the Sample column (ex. E14.5_r1). nCount: number of unique molecules (UMIs), nFeature: number of genes per cell, SCT_snn_res: cell cluster number for various resolutions.

Table S2. Cell-type marker genes for all cell types in the global cell clustering. Marker genes that identify cell types were identified using the Seurat function FindMarkers(). avg_log2FC: log2 fold change, pval: p-value, p_val_adjusted: Benjamini-Hochberg adjusted pvalue, pct.1: percent of observations in given cell types, pct.2: percent of observations in all other cell types.

Table S3. Differentially expressed genes in ACM1 vs. ACM2. Marker genes distinguishing ACM1 from ACM2 were identified using the Seurat function FindMarkers(). avg_log2FC: log2 fold change, pval: p-value, p_val_adjusted: Benjamini-Hochberg adjusted p-value, pct.1: percent of observations in given cell types, pct.2: percent of observations in all other cell types. Genes with a positive log-fold change were enriched in ACM1, while genes with a negative fold change were enriched in ACM2. The second worksheet contains our results for left-right ACM genes identified from a separate study (Feng et al., 2022).

Table S4. Differentially expressed genes across pseudotime for all cell types. Psupertime results for all individual cell types analysed. abs_beta: absolute beta coefficient, a score >0 indicates significant variance across time. *H19* was among or the highest scoring in numerous cell types.

Table S5. Results from enrichment analysis using enrichR against the MGI Mammalian phenotype level 4 2021 database. Genes with significant differential expression across pseudotime per psupertime (abs_beta>0 from Table S4) in more than 2 cell types were tested as one gene list. The p-values were computed using Fisher's exact test, while the combined enrichment scores were calculated by multiplying the log of the p-value with the odds ratio.

Table S6. Endothelial subtype markers from 13wk foetal cardiac endothelial data (McCracken et al., 2022). Marker genes distinguishing various endothelial subtype clusters were identified using the Seurat function FindMarkers(). avg_log2FC: log2 fold change, pval: p-value, p_val_adjusted: Benjamini-Hochberg adjusted p-value, pct.1: percent of observations in given cell types, pct.2: percent of observations in all other cell types. ENDO: endocardial, VEC: valve endothelial cell, VENE: venous vascular endothelial, ARTE: arterial vascular endothelial, VASCE: both venous and arterial.
