# Supplementary material for: Transcriptional dynamics of the murine heart during perinatal development at single-cell resolution
Source: Biol Open. 2026 Sep 11;15(9):bio062493. doi: 10.1242/bio.062493 (PMC13602375; doi:10.1242/bio.062493)
Supplement: Supplementary file 1 [file biolopen-15-062493-s1.pdf]

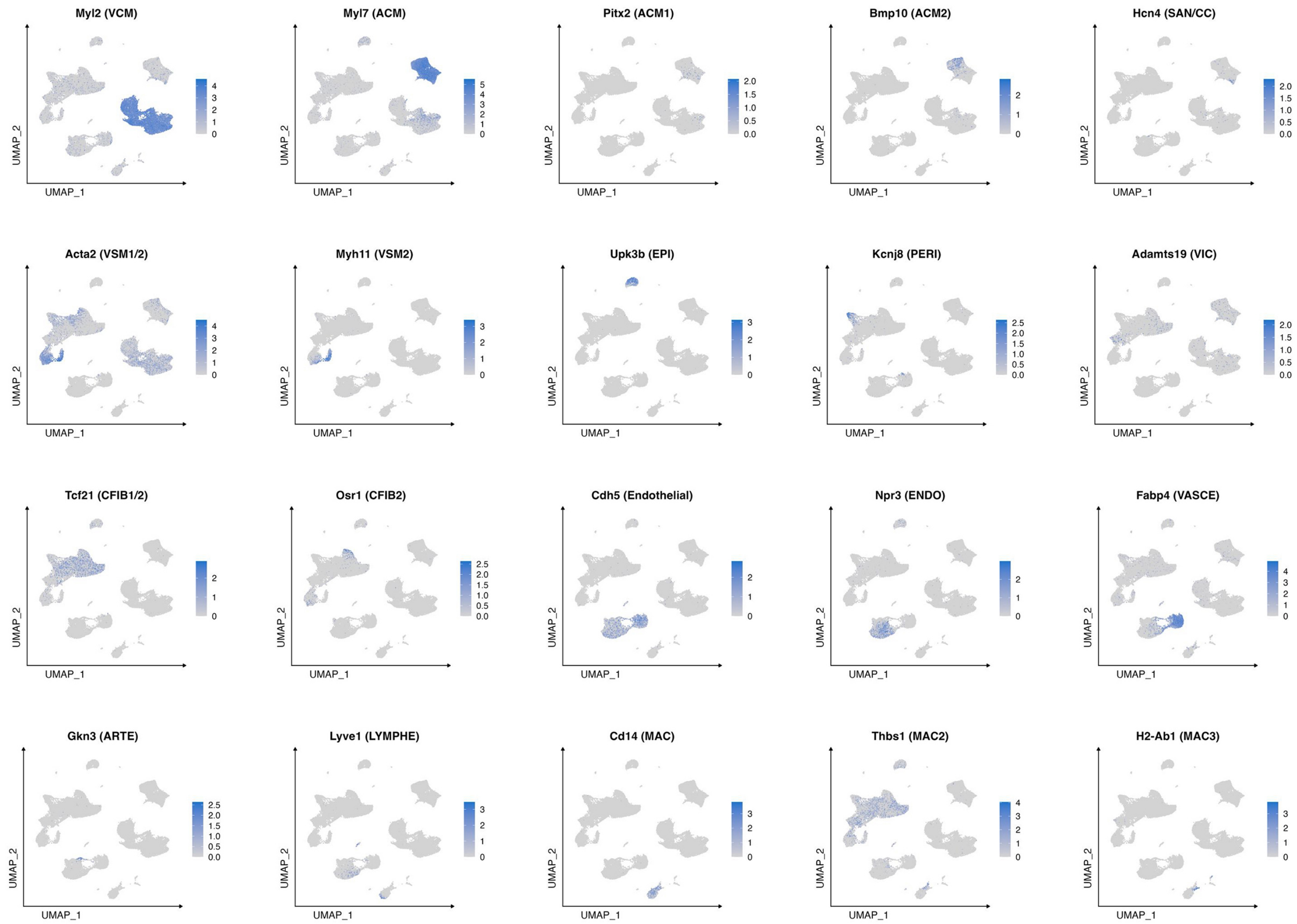

Fig. S1. Feature plots of selected cell type marker genes. *Myl2* (VCM), *Myl7* (ACM), *Pitx2* (ACM1), *Bmp10* (ACM2), *Hcn4* (SAN/CC), *Acta2* (VSM1/2), *Myh11* (VSM2), *Upk3b* (EPI), *Kcnj8* (PERI), *Adamts19* (VIC), *Tcf21* (CFIB1/2), *Osr1* (CFIB2), *Cdh5* (Endothelial), *Npr3* (ENDO), *Fabp4* (VASCE), *Gkn3* (ARTE), *Lyve1* (LYMPHE), *Cd14* (MAC), *Thbs1* (MAC2) and *H2-Ab1* (MAC3).

Biology Open • Supplementary information

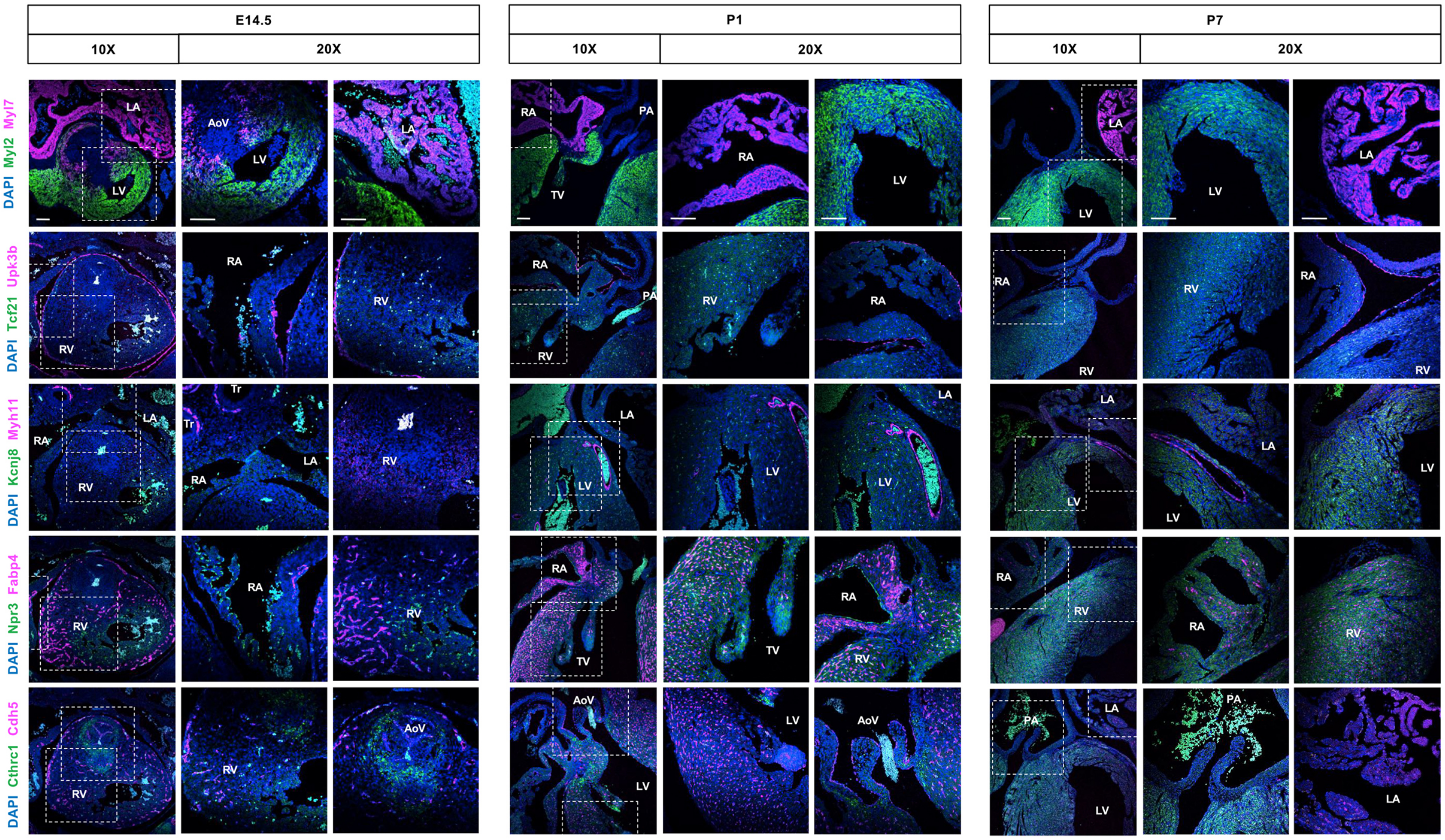

Fig. S2. RNAscope staining for selected marker genes across E14.5, P1, and P7 timepoints at 10x and 20x magnifications. Dotted boxes indicate magnified areas. *Myl2* and *Myl7* stained for ventricular cardiomyocytes and atrial cardiomyocytes respectively. *Tcf21* and *Upk3b* stained for cardiac fibroblast and epicardial cells. *Kcnj8* and *Myh11* stained for pericyte and vascular smooth muscle. *Npr3* and *Fabp4* stained for endocardial cells and vascular endothelial cells. *Cthrc1* and *Cdh5* stained for valve interstitial cells and endothelial cells. DAPI stained for cell nuclei. RA: right atrium, RV: right ventricle, LA: left atrium, LV: left ventricle, TV: tricuspid valve, AoV: aortic valve, PA: pulmonary artery, Tr: trachea. Scale bar: 50 µm. Please note some 10x images (top row P1/P7, second row E14.5/P1 and fourth row E14.5) are replicated from main Figure 2.

Biology Open • Supplementary information

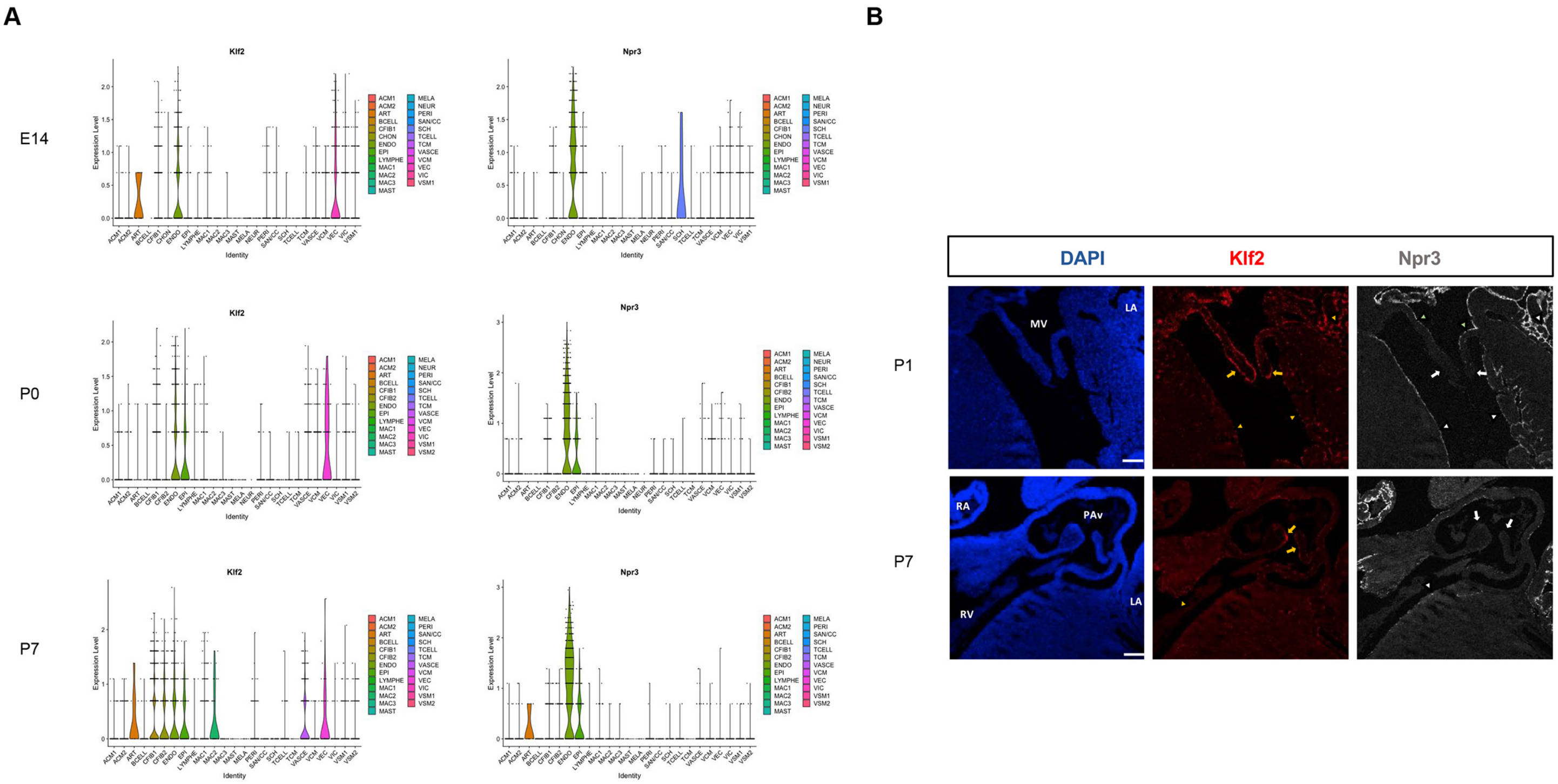

Fig. S3. Npr3 is specific for non-valve endocardium

A) Violin plots showing single cell expression dynamics for *Klf2* and *Npr3* separated by cell type at E14, P0, and P7. Note *Klf2* expression in endocard and VECs, and Npr3 in endocard, but not VECs.

B) RNAScope staining for *Klf2* and *Npr3* RNA expression at P1 and P7, showing strong *Klf2* expression in VECs (yellow arrows) and weak expression in endocardium (yellow arrowheads) (Goddard et al., 2017). *Npr3* is expressed throughout the ventricular and atrial endocardium (white arrowheads), but absent in VECs (white arrows), except for the most proximal mitral valve VECs (green arrowheads) (Zhang et al., 2016). LA = left atrium, MV = mitral valve, PAv = pulmonary artery valve, RA = right atrium, RV = right ventricle. Scale bars: 100 µm.

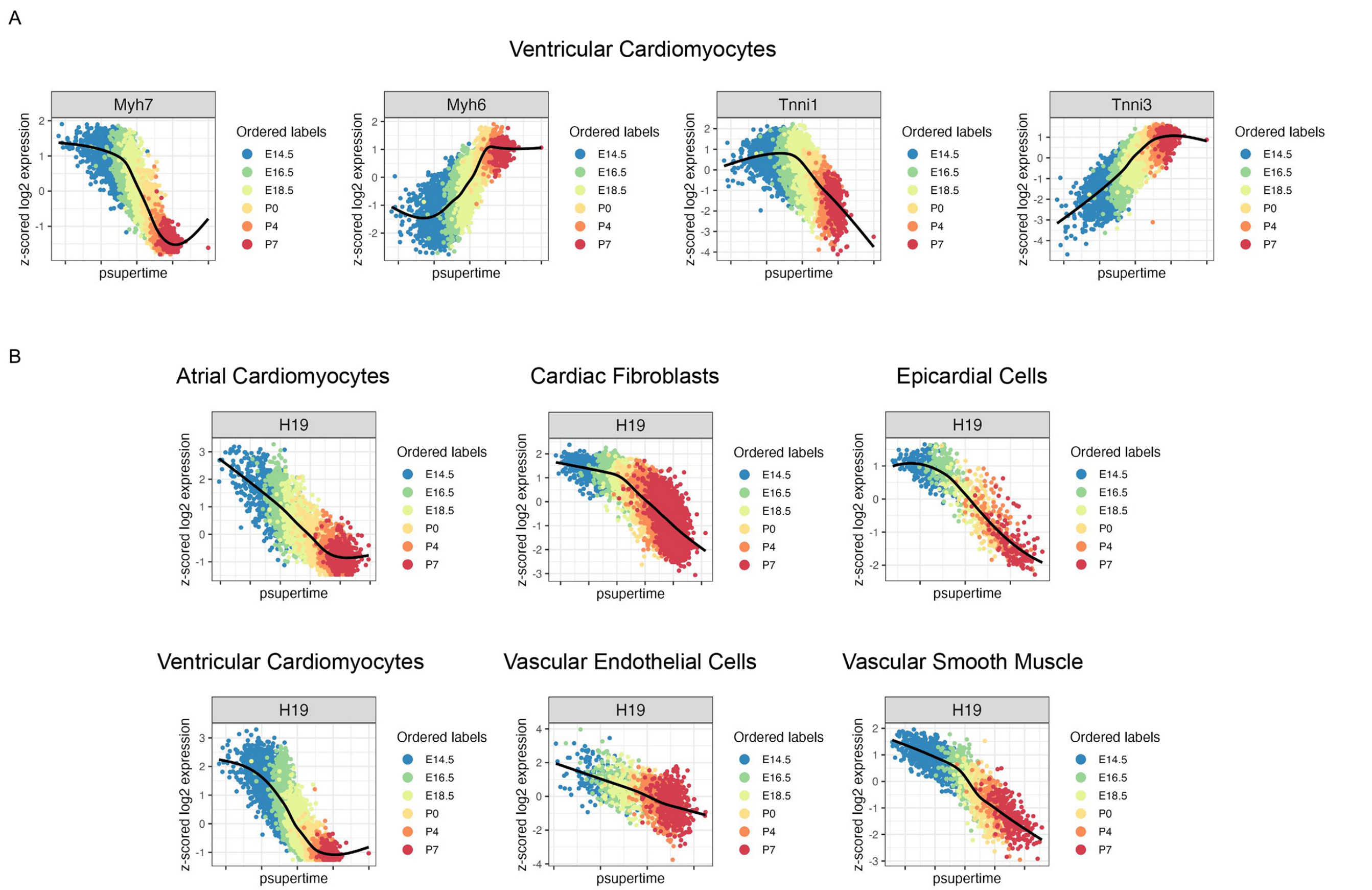

Fig. S4. Temporal gene expression patterns in perinatal mouse hearts. A) Plots of gene expression for *Myh6*, *Myh7*, *Tnni1*, and *Tnni3* against pseudotime scores learned by psupertime in ventricular cardiomyocytes. x-axis: pseudotime score for each cell, y-axis: z-scored log2 gene expression values. All cells are ordered by pseudotime score and coloured by their known time point. A notably sharp drop in expression of *Myh7* can be seen from E18.5 to P7, while *Myh6* steadily increases in expression from E16.5 to P4. This is consistent with known expression patterns.

B) Psupertime plots of gene expression for *H19* against pseudotime scores in multiple cardiac cell types. x-axis: pseudotime score for each cell, y-axis: z-scored log2 gene expression values. *H19* expression decreases consistently across time in cell types of distinct lineages including cardiomyocytes, fibroblasts, epicardial cells, vascular endothelial cells and vascular smooth muscle.

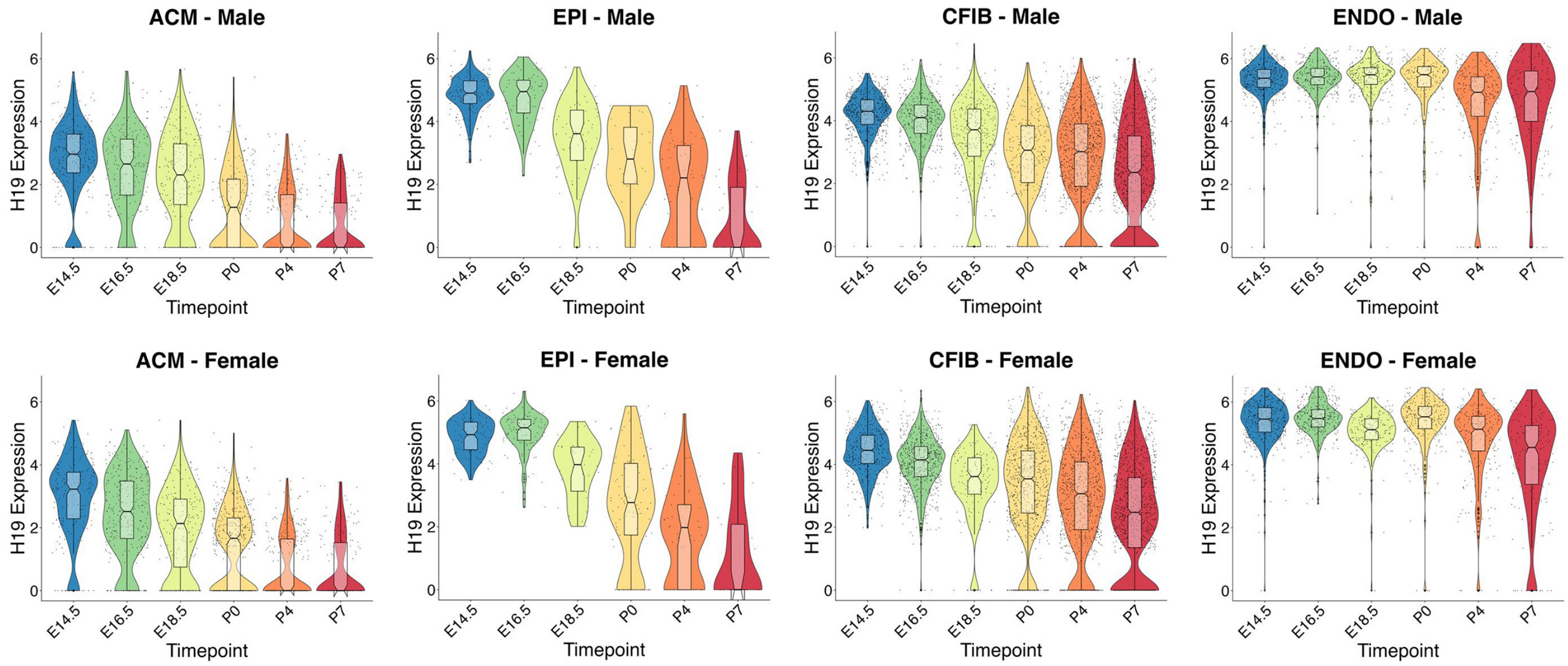

Fig. S5. Seurat violin plots of *H19* gene expression as in Figure 3B for male and female samples in atrial cardiomyocytes, epicardial cells, cardiac fibroblasts and endocardial cells. The expression pattern of *H19* does not appear to be sex-specific.

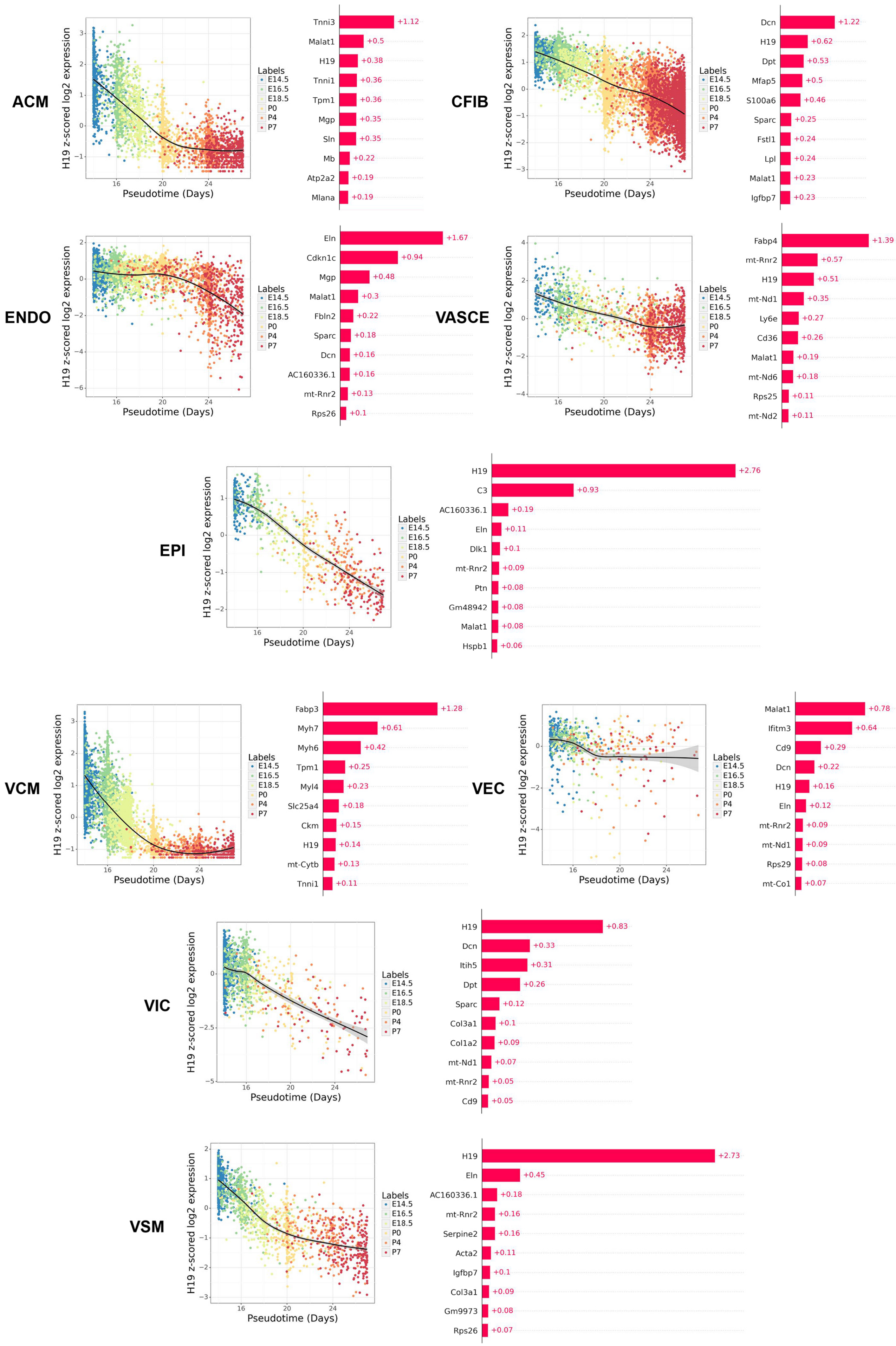
ACM
H19 z-scored log2 expression
Pseudotime (Days)
Labels
E14.5
E16.5
E18.5
P0
P4
P7
Tnni3 +1.12
Malat1 +0.5
H19 +0.38
Tnni1 +0.36
Tpm1 +0.36
Mgp +0.35
Sln +0.35
Mb +0.22
Atp2a2 +0.19
Mlana +0.19
CFIB
Dcn +1.22
H19 +0.62
Dpt +0.53
Mfap5 +0.5
S100a6 +0.46
Sparc +0.25
Fstl1 +0.24
Lpl +0.24
Malat1 +0.23
Igfbp7 +0.23
ENDO
Eln +1.67
Cdkn1c +0.94
Mgp +0.48
Malat1 +0.3
Fbln2 +0.22
Sparc +0.18
Dcn +0.16
AC160336.1 +0.16
mt-Rnr2 +0.13
Rps26 +0.1
VASCE
Fabp4 +1.39
mt-Rnr2 +0.57
H19 +0.51
mt-Nd1 +0.35
Ly6e +0.27
Cd36 +0.26
Malat1 +0.19
mt-Nd6 +0.18
Rps25 +0.11
mt-Nd2 +0.11
EPI
H19 +2.76
C3 +0.93
AC160336.1 +0.19
Eln +0.11
Dlk1 +0.1
mt-Rnr2 +0.09
Ptn +0.08
Gm48942 +0.08
Malat1 +0.08
Hspb1 +0.06
VCM
Fabp3 +1.28
Myh7 +0.61
Myh6 +0.42
Tpm1 +0.25
Myl4 +0.23
Slc25a4 +0.18
Ckm +0.15
H19 +0.14
mt-Cytb +0.13
Tnni1 +0.11
VEC
Malat1 +0.78
Ifitm3 +0.64
Cd9 +0.29
Dcn +0.22
H19 +0.16
Eln +0.12
mt-Rnr2 +0.09
mt-Nd1 +0.09
Rps29 +0.08
mt-Co1 +0.07
VIC
H19 +0.83
Dcn +0.33
Itih5 +0.31
Dpt +0.26
Sparc +0.12
Col3a1 +0.1
Col1a2 +0.09
mt-Nd1 +0.07
mt-Rnr2 +0.05
Cd9 +0.05
VSM
H19 +2.73
Eln +0.45
AC160336.1 +0.18
mt-Rnr2 +0.16
Serpine2 +0.16
Acta2 +0.11
Igfbp7 +0.1
Col3a1 +0.09
Gm9973 +0.08
Rps26 +0.07

Fig. S6. Plots from Sceptic pseudotime analysis. Left for each cell type: Plots of gene expression for *H19* against pseudotime scores derived by Sceptic. As with psupertime, the timepoints were provided as inputs but were also given an approximate “real time” value, in this case days (E14.5=14d, E16.5=16d, E18.5=18d, P0=20d, P4=24d and P7=28d). The resulting plots were comparable to those obtained by psupertime. Right for each cell type: Bar plots of absolute mean SHAP (SHapley Additive exPlanations) values, indicating which genes most influenced Sceptic’s model for calculating pseudotime scores. *H19* was the top hit for EPI, VIC and VSM, and within the top 3 for ACM, CFIB and VASCE, but was not within the top results for ENDO.

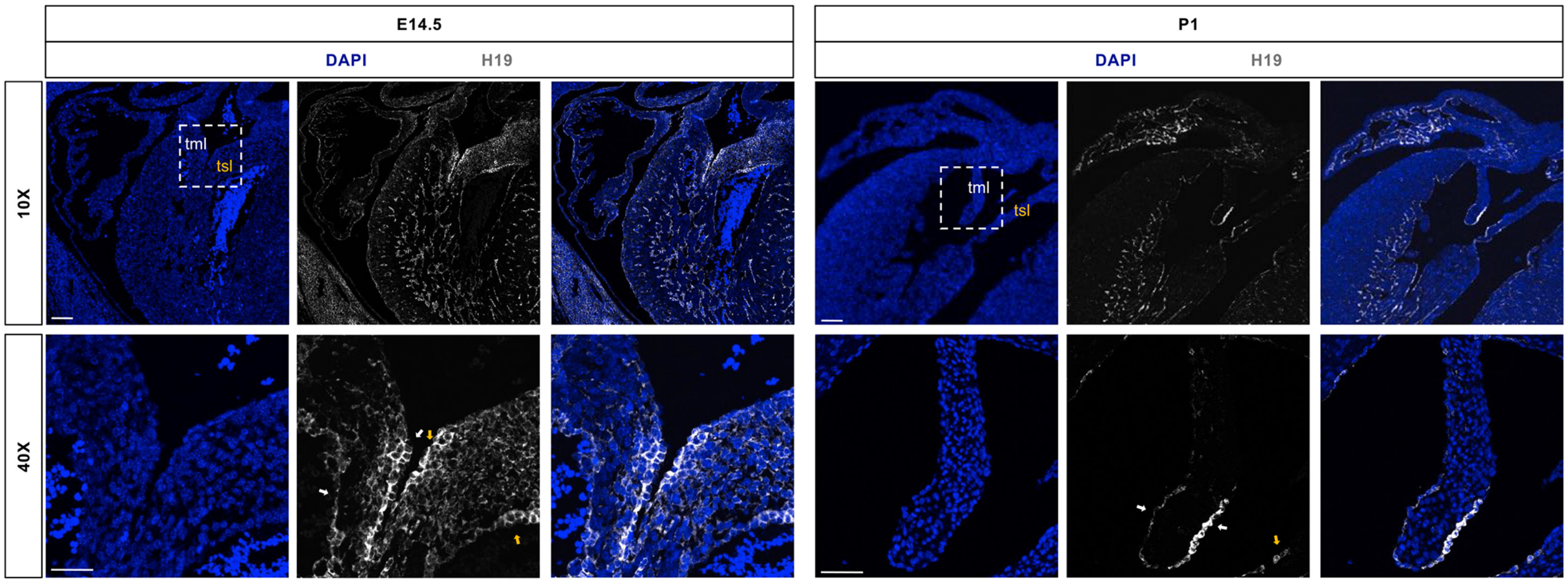

Fig. S7. RNAScope staining for *H19* in the tricuspid valves at E14.5 and P1. At E14.5, *H19* is equally expressed in the tricuspid mural valve leaflet (tml, white arrows) and tricuspid septal valve leaflet (tsl, yellow arrows). Note the strong expression on the coaptation sides of both leaflets at E14.5. At P1, *H19* expression becomes more confined to the distal VECs, in particular at the tricuspid mural valve leaflet coaptation side. Scale bars: 100 µm. Please note the 10x E14.5 and P1 images are replicated from main Figure 2 (fifth row), and the 40x E14.5 and P1 images are replicated from main Figure 4E.

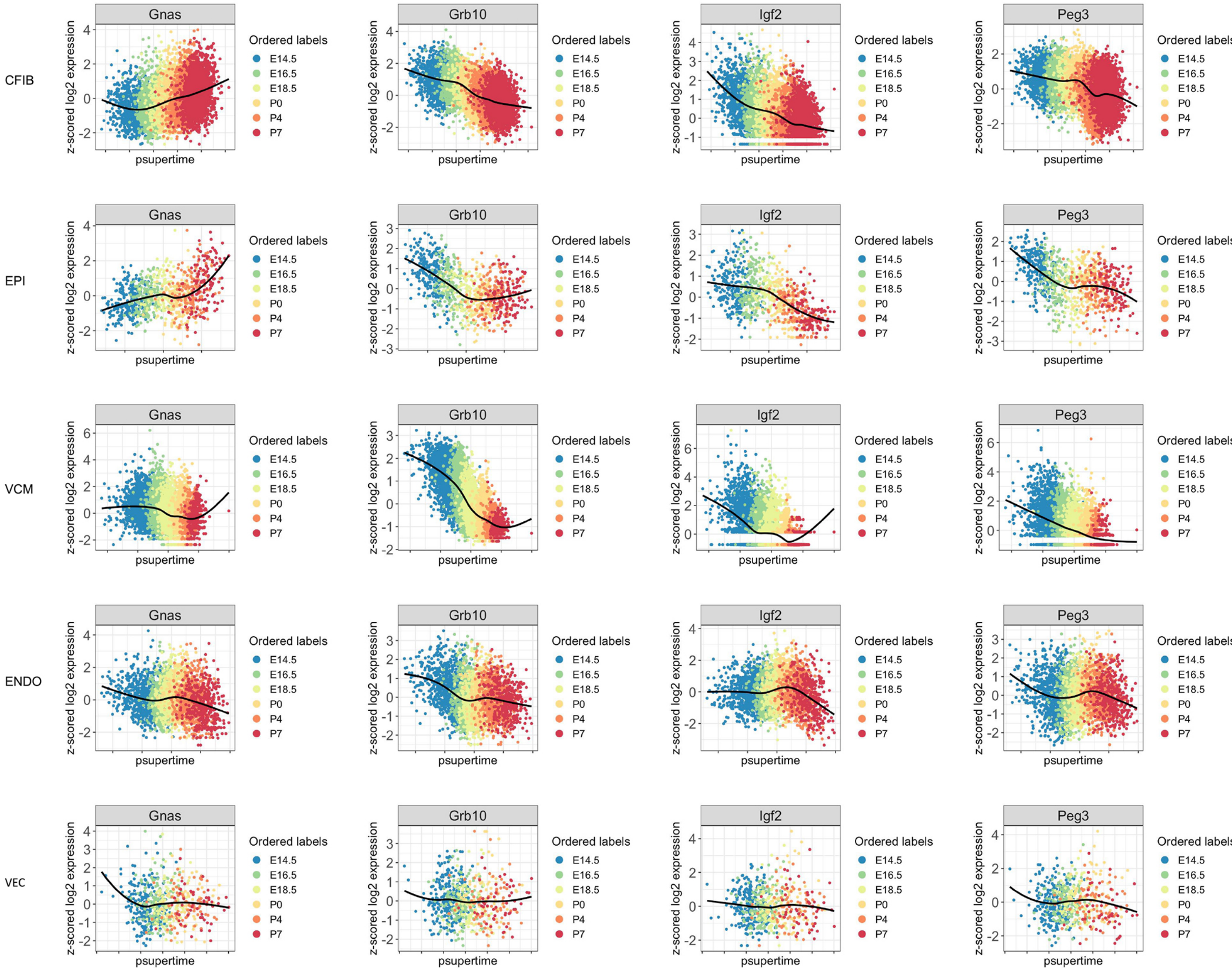

Fig. S8. Psupertime plots of gene expression for *Gnas, Grb10, Igf2* and *Peg3* against pseudotime scores in multiple cardiac cell types. x-axis: pseudotime score for each cell, y-axis: z-scored log2 gene expression values. These genes were identified from the imprinted gene sets found by EnrichR to be highly enriched over maturation (**Table S5**). In most cell types a notable change in expression of these genes can be seen, often decreasing over time. However, comparatively less variation in expression is seen in endocardial and especially valve endothelial cells.

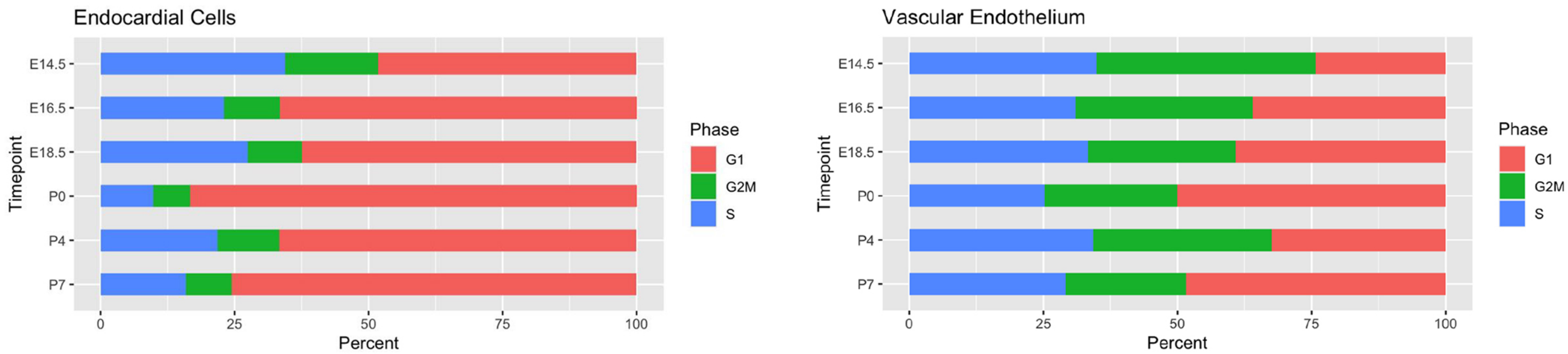

Fig. S9. Bar plots of cell cycle phase per timepoint. For each timepoint, the percentage of cells in G1, G2/M, and S phase (assigned by Seurat) is visualized for endocardial cells (left side) and vascular endothelial cells (right side).

Biology Open • Supplementary information

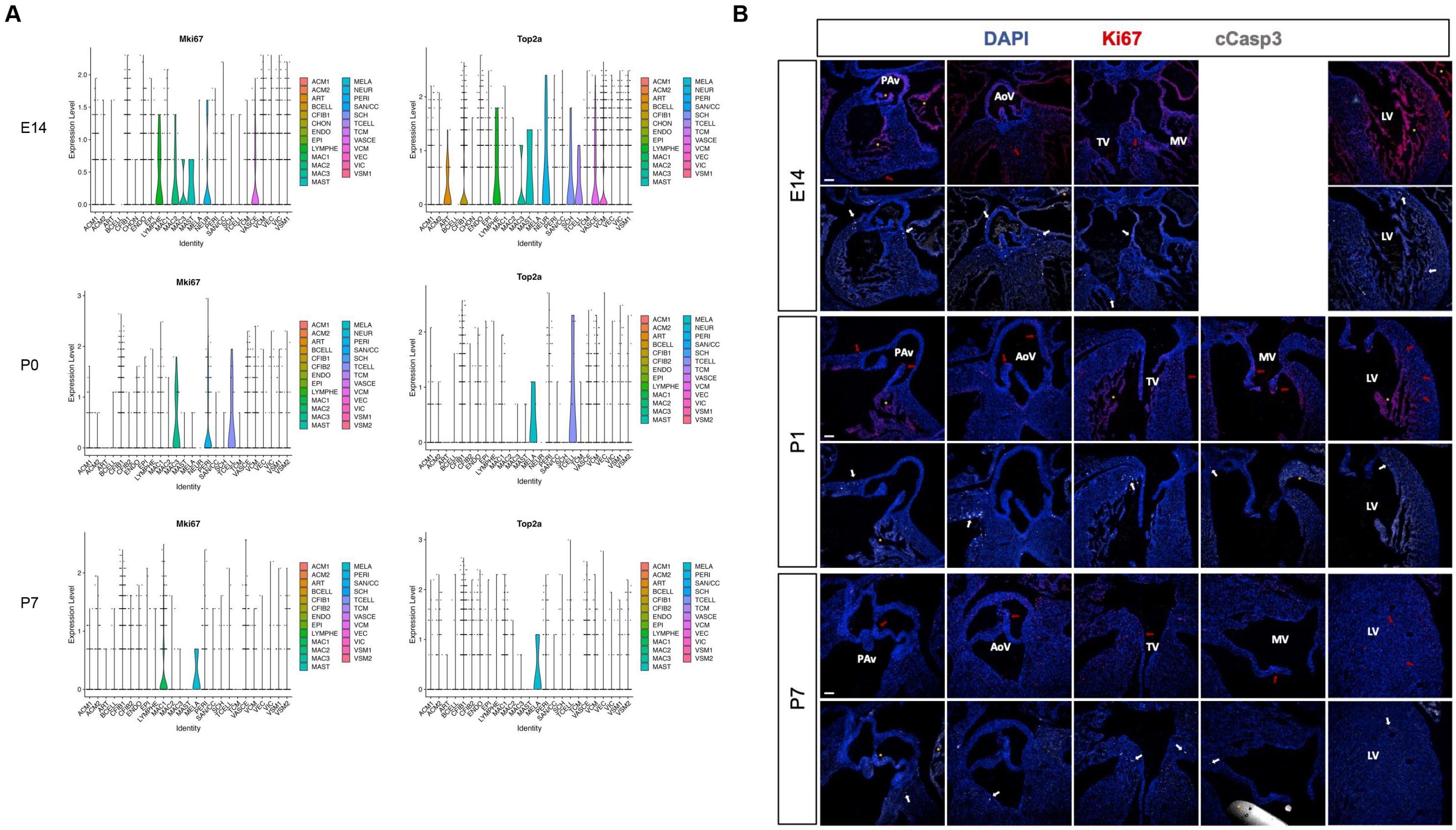

Fig. S10. Cell proliferation and apoptosis marker expression in perinatal mouse hearts.

A) Seurat violin plots for proliferation markers *Top2a* and *Ki67* at E14, P0 and P7 grouped by cell type. No changes in expression over time were seen in endocardial (ENDO), valve endothelial (VEC) or valve interstitial (VIC) cells.

B) Immunostaining for Ki67 and cCasp3 at E14, P1, and P7. AoV = aortic valve, PAv = pulmonary artery valve, TV= tricuscpid valve, MV = mitral valve, LV = left ventricle. Red arrows indicate Ki67+ cells, white arrows indicate cCasp3+ cells, yellow asterisks indicate background signal (particularly in atrial and trabecular myocardium). Note the clusters of proliferative and apoptotic cells in the base of the four major valves, and presence of Ki67+ cells but near-absence of cCasp+ cells in the valve leaflets. Proliferation in the left ventricular wall gradually decreases between E14.5 and P7. Scale bars are 100 µm.

Biology Open • Supplementary information

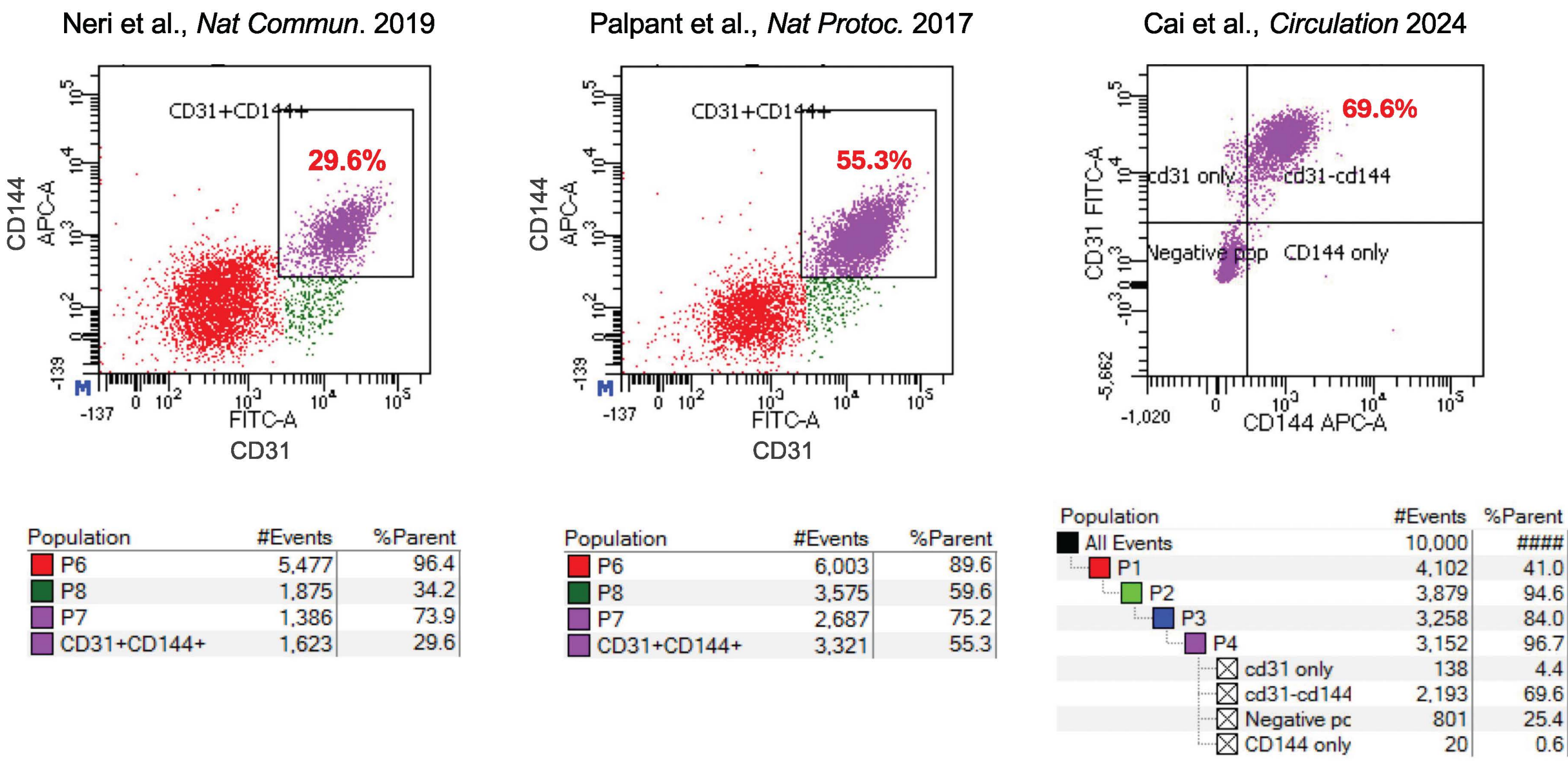

Population	#Events	%Parent
P6	5,477	96.4
P8	1,875	34.2
P7	1,386	73.9
CD31+CD144+	1,623	29.6

Population	#Events	%Parent
P6	6,003	89.6
P8	3,575	59.6
P7	2,687	75.2
CD31+CD144+	3,321	55.3

Population	#Events	%Parent
All Events	10,000	####
P1	4,102	41.0
P2	3,879	94.6
P3	3,258	84.0
P4	3,152	96.7
cd31 only	138	4.4
cd31-cd144	2,193	69.6
Negative pc	801	25.4
CD144 only	20	0.6

Fig. S11. Flow cytometry results for various hiPSC-derived endothelial cell differentiation protocols. Three differentiation protocols (Neri et al., 2019; Palpant et al., 2017; Cai et al., 2024) were initially compared to determine which protocol was most efficient at generating endothelial cells, as measured by the percentage of cells double-positive for CD31 (PECAM1) and CD144 (CDH5).

Biology Open • Supplementary information

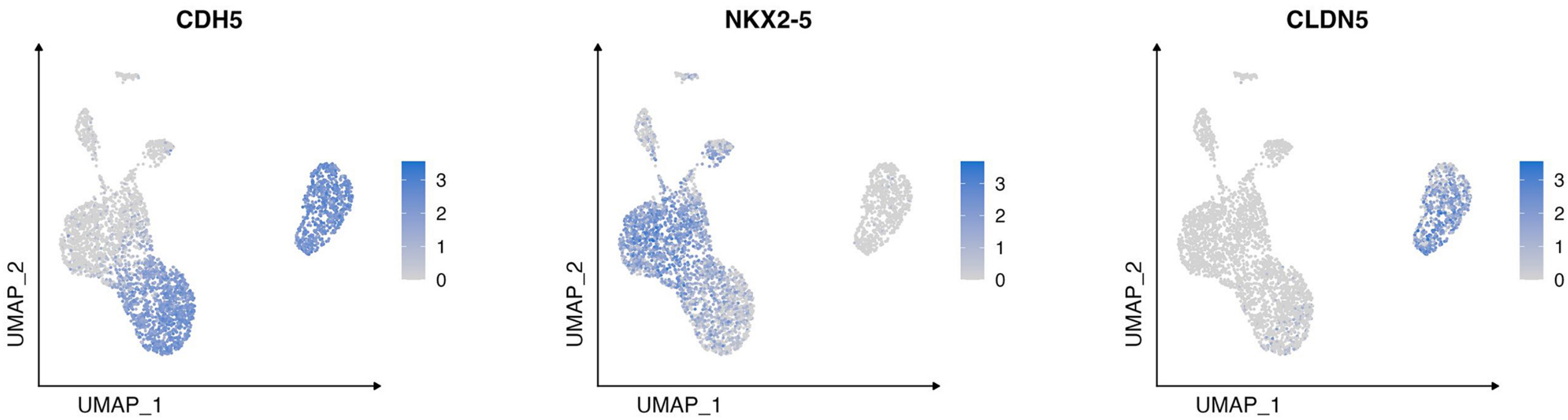

Fig. S12. Feature plots for hiPSC-derived cells (Mikryukov et al., 2021). *CDH5* expression (left plot) labels two distinct pan-endothelial cell clusters consisting of *NKX2-5+/CLDN5-* endocardial cells and *NKX2-5-/CLDN5+* vascular cells (middle and right plots), validating *CLDN5* as a marker to distinguish hiPSC-derived endothelial subtypes.

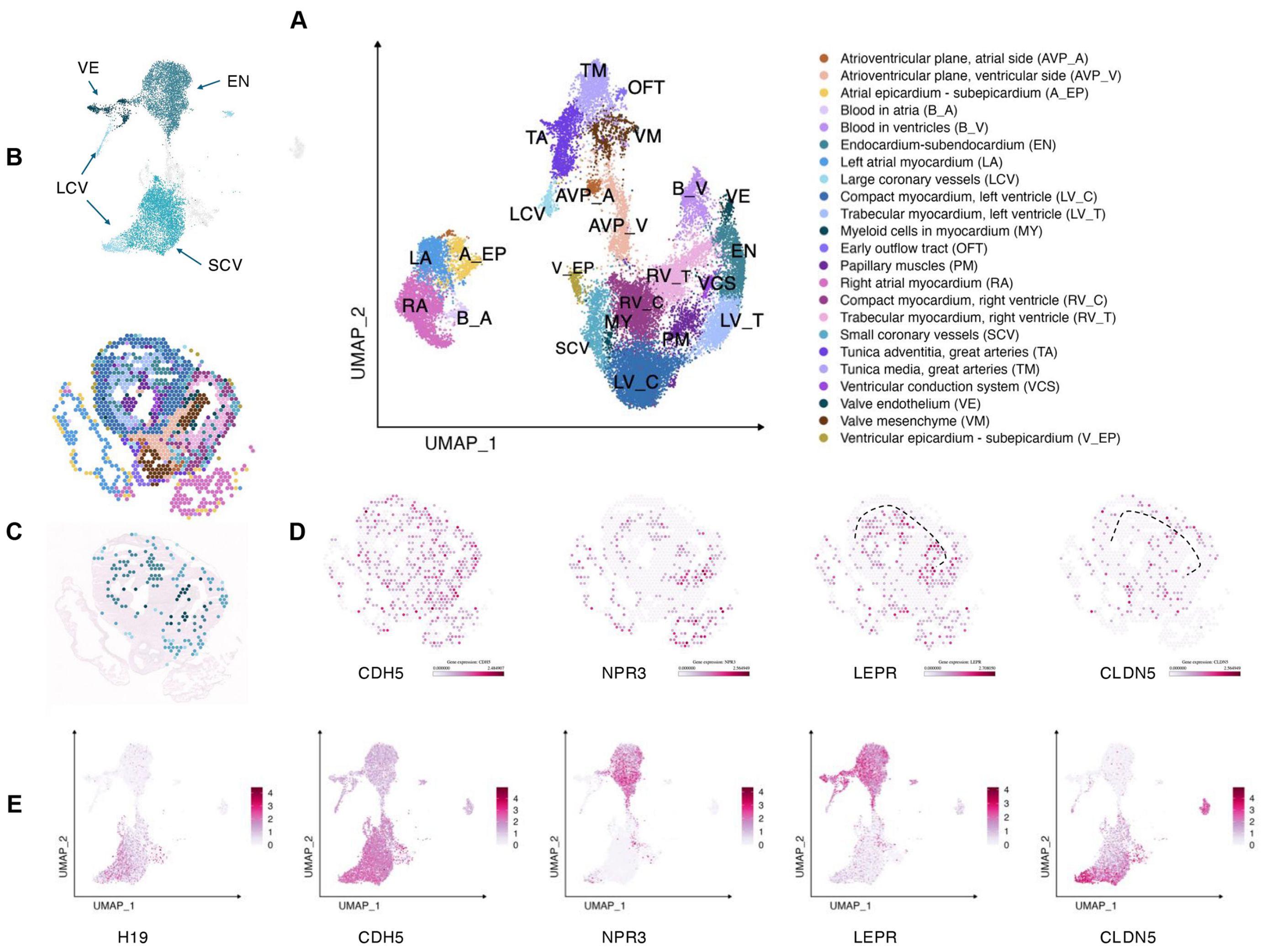

Fig. S13. Spatial data validation of endothelial cell subtype specificity for *LEPR* and *CLDN5*.

A) Full dataset UMAP from Lázár et al. featuring 23 cell types, replicating original clustering and colouring (Lázár et al., 2025 Fig. 1b) and reproduced using a publicly available R object.

B) UMAP of endothelial-subsetted cells from Lázár et al. including endocardial cells (EN), valve endothelial cells (VE), small coronary vessel endothelium (SCV) and large coronary vessel endothelium (LCV), also replicating original clustering (Lázár et al., 2025 Fig. 5a) and reproduced using a publicly available R object. Colouring for the endothelial cell subtypes (varying shades of blue) corresponds to the UMAP in **A)**.

C) Spatial clustering showing all cell types (top) and endothelial cell types only (bottom), taken from Lázár et al.'s interactive viewer (https://hdcaheart.serve.scilifelab.se). Colouring corresponds to the UMAP in A).

Biology Open • Supplementary information

D) Spatial images for *CDH5*, *NPR3*, *LEPR*, and *CLDN5* in an 8-week foetal heart taken from Lázár et al.'s interactive viewer. The dotted line indicates the border between the trabecular endocard and ventricular coronary vasculature. *LEPR* and *CLDN5* show distinct spatial localization in respectively the endocardial and coronary vessel regions.

E) Feature plots for genes of interest in endothelial-subsetted cells corresponding to the UMAP in **B).** LEPR is strongly expressed in endocard and valve endothelium, while *CLDN5* is exclusively expressed in coronary vessels. Note that *H19* is more highly expressed in human embryonic vascular cells than endocardial cells, contrary to mouse data (see Fig. S12).

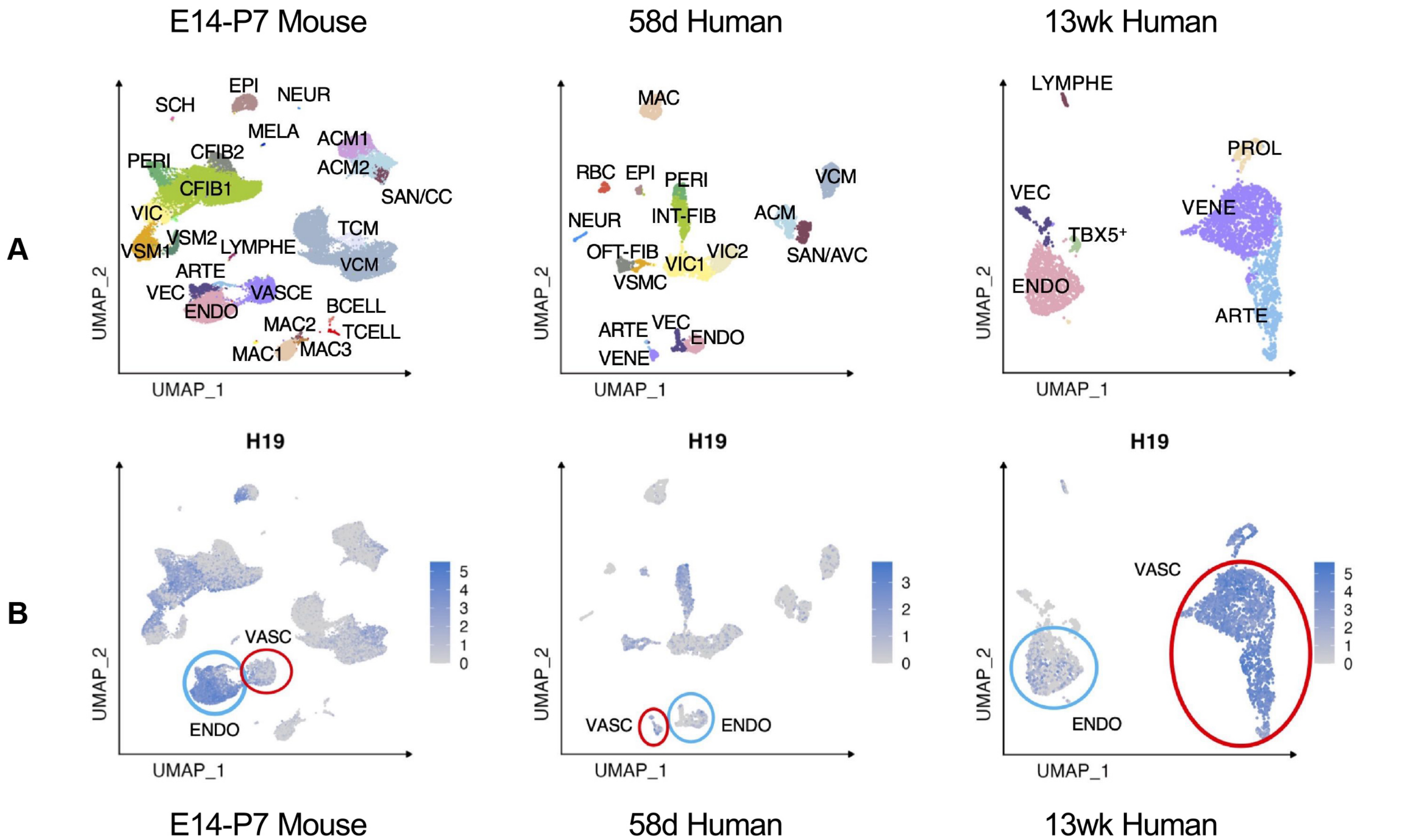

Fig. S14. *H19* shows species-specific expression patterns within subtypes of cardiac endothelial cells

A) UMAPs from our E14-P7 mouse data as in Figure 1 and re-analysed single cell datasets from Knight-Schrijver et al (58d human foetal whole heart) and McCracken et al (13wk human foetal endothelial cells pre-sorted for CDH5).

B) Respective feature plots for H19 expression *in vivo*. In perinatal mouse hearts, H19 is more strongly expressed in endocardial cells compared to vascular cells. This pattern however is reversed in human embryonic / foetal hearts, where *H19* is instead strongly expressed in vascular cells and very lowly in endocardial cells.

Biology Open • Supplementary information

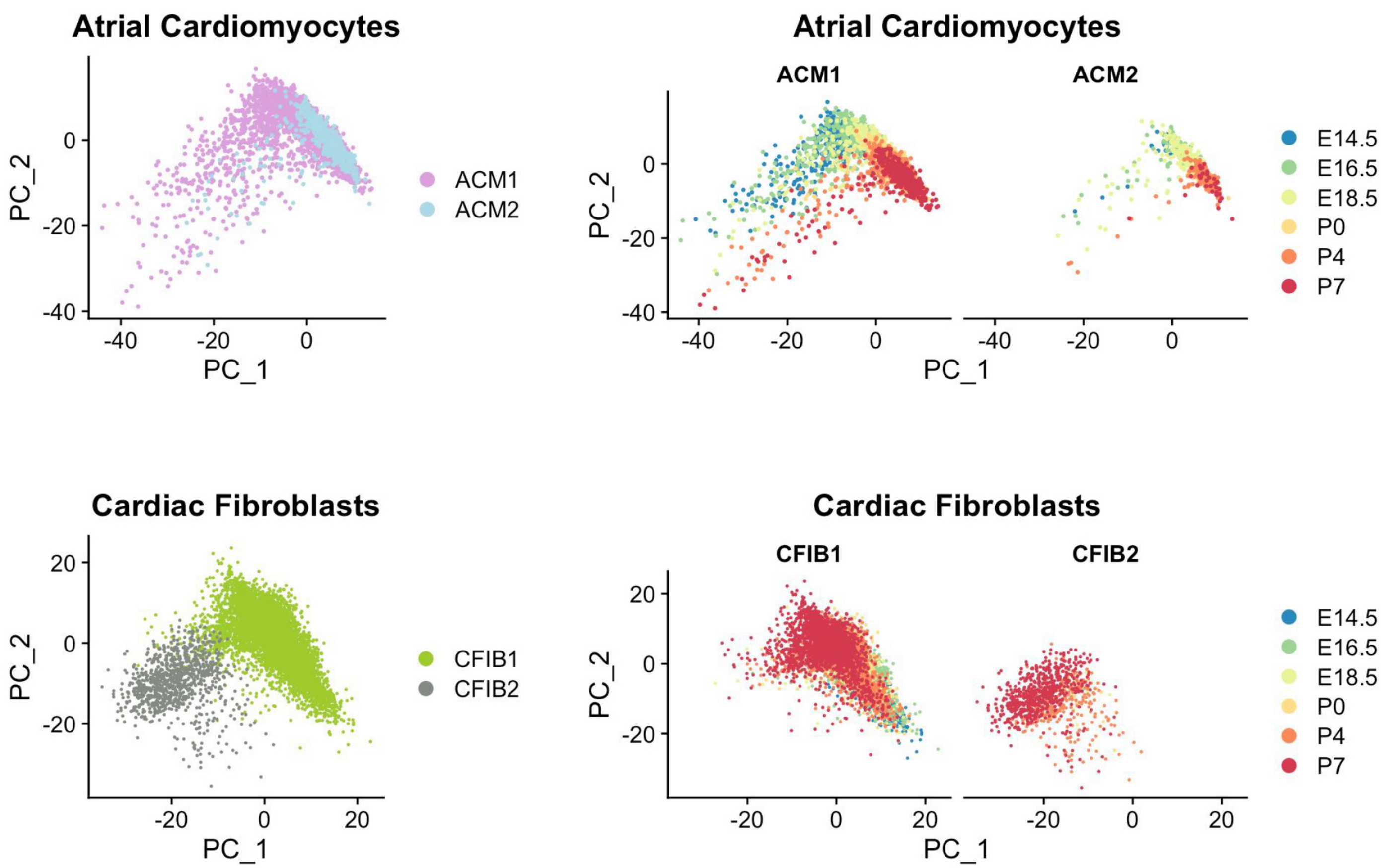

Fig. S15. PCA plots of all atrial cardiomyocytes and all cardiac fibroblasts. The ACM1 and ACM2 clusters combine to form a continuous trajectory, while CFIB1 and CFIB2 appear to be divergent.

Table S1. Metadata information for all cells in final single-cell dataset.

Fastq files for E14.5 samples were previously published (Wünnemann et al.) and reanalysed from raw data using the same pipeline used for all other samples. Six replicates were used per time point for a total of 36 mice as indicated in the Sample column (ex. E14.5_r1). nCount: number of unique molecules (UMIs), nFeature: number of genes per cell, SCT_snn_res: cell cluster number for various resolutions.

Available for download at
https://journals.biologists.com/bio/article-lookup/doi/10.1242/bio.062493#supplementary-data

Table S2. Cell-type marker genes for all cell types in the global cell clustering.

Marker genes that identify cell types were identified using the Seurat function FindMarkers(). avg_log2FC: log2 fold change, pval: p-value, p_val_adjusted: Benjamini-Hochberg adjusted p-value, pct.1: percent of observations in given cell types, pct.2: percent of observations in all other cell types.

Available for download at
https://journals.biologists.com/bio/article-lookup/doi/10.1242/bio.062493#supplementary-data

Table S3. Differentially expressed genes in ACM1 vs. ACM2. Marker genes distinguishing ACM1 from ACM2 were identified using the Seurat function FindMarkers(). avg_log2FC: log2 fold change, pval: p-value, p_val_adjusted: Benjamini-Hochberg adjusted p-value, pct.1: percent of observations in given cell types, pct.2: percent of observations in all other cell types. Genes with a positive log-fold change were enriched in ACM1, while genes with a negative fold change were enriched in ACM2. The second worksheet contains our results for left-right ACM genes identified from a separate study (Feng et al., 2022).

Available for download at
https://journals.biologists.com/bio/article-lookup/doi/10.1242/bio.062493#supplementary-data

Table S4. Differentially expressed genes across pseudotime for all cell types.

Psupertime results for all individual cell types analysed. abs_beta: absolute beta coefficient, a score >0 indicates significant variance across time. *H19* was among or the highest scoring in numerous cell types.

Available for download at
https://journals.biologists.com/bio/article-lookup/doi/10.1242/bio.062493#supplementary-data

Table S5. Results from enrichment analysis using enrichR against the MGI Mammalian phenotype level 4 2021 database.

Genes with significant differential expression across pseudotime per psupertime (abs_beta>0 from Table S4) in more than 2 cell types were tested as one gene list. The p-values were computed using Fisher's exact test, while the combined enrichment scores were calculated by multiplying the log of the p-value with the odds ratio.

Available for download at
https://journals.biologists.com/bio/article-lookup/doi/10.1242/bio.062493#supplementary-data

Table S6. Endothelial subtype markers from 13wk foetal cardiac endothelial data (McCracken et al., 2022). Marker genes distinguishing various endothelial subtype clusters were identified using the Seurat function FindMarkers(). avg_log2FC: log2 fold change, pval: p-value, p_val_adjusted: Benjamini-Hochberg adjusted p-value, pct.1: percent of observations in given cell types, pct.2: percent of observations in all other cell types. ENDO: endocardial, VEC: valve endothelial cell, VENE: venous vascular endothelial, ARTE: arterial vascular endothelial, VASCE: both venous and arterial.

Available for download at
https://journals.biologists.com/bio/article-lookup/doi/10.1242/bio.062493#supplementary-data

Table S7. Primers used for RT-qPCR

Gene	Primers
HPRT1	CCTGGCGTCGTGATTAGTGA (F)
	CGAGCAAGACGTTCAGTCCT (R)
RPL13A	GTCGTACGCTGTGAAGGCAT (F)
	GTGGGGCAGCATACCTCG (R)
H19	TCAAGCCTGGGCCTTTGAAT (F)
	GGCTGATGAGGTCTGGTTCC (R)
CD31	TGCCGTGGAAAGCAGATACT (F)
	TAAGAACCGGCAGCTTAGCC (R)
CDH5	CTTCACCCAGACCAAGTACACA (F)
	GGCTCATGTATCGGAGGTCG (R)
TIE1	GCTCCCCATCCTCTTCTTGG (F)
	CCAGACACGCAAGTCAGGAA (R)
NPR3	AGGGTTTGCACACGTCCATC (F)
	GCTCGCACACATGATCACCA (R)
LEPR	GTTCCTGGGCACAAGGACTTA (F)
	AGGCTCCAAAAGAAGAGGACC (R)
CLDN5	AAGTGTACGACTCGGTGCTG F (F)
	AAACAGGTAGAGCACGCCTC R (R)
TWIST1	GCCGGAGACCTAGATGTCATT (F)
	TTTTAAAAGTGCGCCCCACG (R)

Biology Open • Supplementary information
